# Characterization of the Contradictory Chromatin Signatures at the 3′ Exons of Zinc Finger Genes

**DOI:** 10.1371/journal.pone.0017121

**Published:** 2011-02-15

**Authors:** Kimberly R. Blahnik, Lei Dou, Lorigail Echipare, Sushma Iyengar, Henriette O'Geen, Erica Sanchez, Yongjun Zhao, Marco A. Marra, Martin Hirst, Joseph F. Costello, Ian Korf, Peggy J. Farnham

**Affiliations:** 1 Genetics Graduate Group, University of California Davis, Davis, California, United States of America; 2 Genome Center, University of California Davis, Davis, California, United States of America; 3 Department of Computer Science, University of California Davis, Davis, California, United States of America; 4 Genome Sciences Centre, BC Cancer Agency, Vancouver, Canada; 5 Department of Neurosurgery, Brain Tumor Research Center, Helen Diller Family Comprehensive Cancer Center, University of California San Francisco, San Francisco, California, United States of America; 6 Department of Molecular and Cellular Biology, University of California Davis, Davis, California, United States of America; 7 Department of Biochemistry and Molecular Biology, Norris Comprehensive Cancer Center, University of Southern California, Los Angeles, California, United States of America; Wellcome Trust Centre for Stem Cell Research, United Kingdom

## Abstract

The H3K9me3 histone modification is often found at promoter regions, where it functions to repress transcription. However, we have previously shown that 3′ exons of zinc finger genes (ZNFs) are marked by high levels of H3K9me3. We have now further investigated this unusual location for H3K9me3 in ZNF genes. Neither bioinformatic nor experimental approaches support the hypothesis that the 3′ exons of ZNFs are promoters. We further characterized the histone modifications at the 3′ ZNF exons and found that these regions also contain H3K36me3, a mark of transcriptional elongation. A genome-wide analysis of ChIP-seq data revealed that ZNFs constitute the majority of genes that have high levels of both H3K9me3 and H3K36me3. These results suggested the possibility that the ZNF genes may be imprinted, with one allele transcribed and one allele repressed. To test the hypothesis that the contradictory modifications are due to imprinting, we used a SNP analysis of RNA-seq data to demonstrate that both alleles of certain ZNF genes having H3K9me3 and H3K36me3 are transcribed. We next analyzed isolated ZNF 3′ exons using stably integrated episomes. We found that although the H3K36me3 mark was lost when the 3′ ZNF exon was removed from its natural genomic location, the isolated ZNF 3′ exons retained the H3K9me3 mark. Thus, the H3K9me3 mark at ZNF 3′ exons does not impede transcription and it is regulated independently of the H3K36me3 mark. Finally, we demonstrate a strong relationship between the number of tandemly repeated domains in the 3′ exons and the H3K9me3 mark. We suggest that the H3K9me3 at ZNF 3′ exons may function to protect the genome from inappropriate recombination rather than to regulate transcription.

## Introduction

It is becoming increasingly clear that understanding human health and disease requires a detailed knowledge of both the genetic and epigenetic variations in the human population. Epigenomes are characterized by methylated DNA and certain modified histones. The patterns of methylated DNA and modified histones can vary greatly from cell type to cell type and large changes within a cell type have also been observed when comparing normal to diseased tissue [1], [2], [3], [4], [5], [6], [7], [8]. Ongoing research is currently investigating how epigenomes differ from individual to individual when comparing the same cell type collected from many different people (http://www.roadmapepigenomics.org/). Six different modified histones have been chosen as the focus of a large amount of research due to their association with specific gene structures. For example, H3K9Ac, H3K4me3, H3K27me3, and H3K9me3 are histone modifications that can be found at promoter regions, whereas other modified histones are associated with enhancer regions (e.g H3K4me1) or with transcribed units (e.g. H3K36me3).

As indicated above, H3K27me3 and H3K9me3 are often found at promoter regions. In fact, initial studies of H3K27me3 and H3K9me3 using ChIP-chip and promoter arrays identified large sets of promoters that were distinguished by these two marks, often in a cell type-specific pattern [9], [10], [11], [12]. However, when studies were expanded to ChIP-chip using genomic tiling arrays and then to genome-wide ChIP-seq, it became clear that H3K27me3 and H3K9me3 were not only found at promoter regions but that these marks could also spread over larger genomic regions. For H3K27me3, the spreading patterns are generally found over HOX gene clusters [9]. Analysis of the H3K9me3 mark is more complicated. H3K9me3 has been found to cover various repetitive regions, such as centromeres, transposons, and tandem repeats [13], [14], [15]. In addition, we have previously shown that the 3′ exons of many zinc finger genes (ZNFs) are covered by H3K9me3 [10]. The purpose of this current study is to further investigate the set of ZNF 3′ exons that are highly enriched for the H3K9me3 mark.

## Results

**Figure 1:**
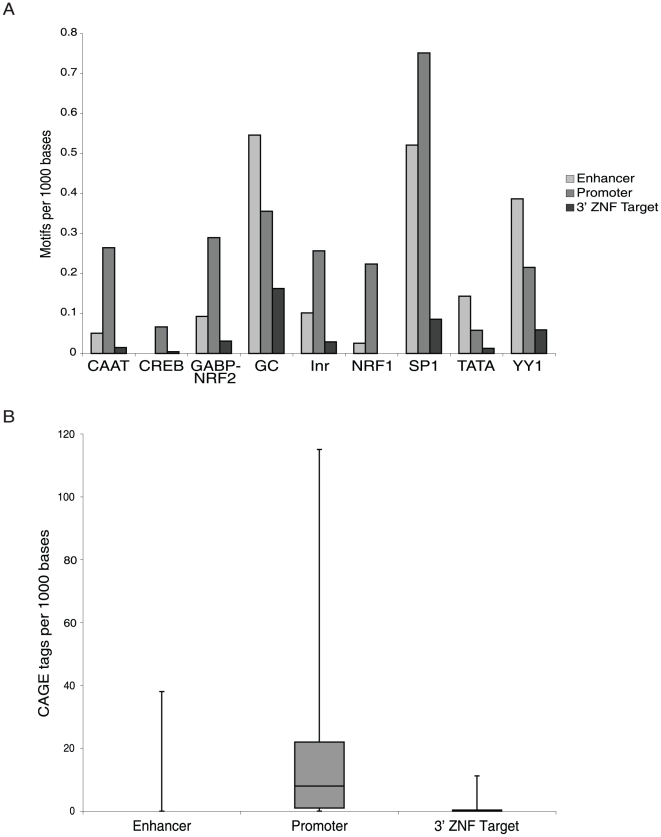
ZNF 3′ ends are not enriched for promoter motifs or CAGE tags. (A) The 3′ ends of the ZNFs bound by H3K9me3, as well as a 1kb region centered on the start site (promoter) and a 1 kb region located 10 kb upstream (enhancer) of the same genes were searched in forward and reverse orientations for the sequence of nine motifs previously found to be enriched in promoter regions [16]. Since the sizes of the H3K9me3 regions varied from site to site, the data was normalized such that the motif counts are reported as the number of motifs found per 1000 base pairs. (B) The number of CAGE tags corresponding to the regions of the 3′ ends of the ZNFs bound by H3K9me3, as well as a 1 kb region centered on the start site (promoter) and a 1 kb region located 10 kb upstream (enhancer) of the same genes was determined; the data was normalized as described in Figure 1A.

The 3′ ends of ZNF genes do not have characteristics of promoter regions. As indicated above, H3K9me3 is often associated with promoter regions but our previous studies showed that it specifically covers the 3′ exons of many ZNF genes. Because H3K9me3 is not generally associated with the 3′ ends of genes, we reasoned that it was possible that the 3′ ends of the ZNF genes may harbor previously unidentified promoters. To investigate this hypothesis, we first performed a bioinformatic analysis. Others [16] have previously identified a set of motifs that are highly enriched in human promoters. If the 3′ ends of the ZNF genes are previously unidentified promoters, then we would expect the known promoter motifs to be enriched in these regions. We extracted the target region for each ZNF 3′ end on chr19 that was identified using ChIP-seq of hES cells as being enriched for H3K9me3 (121 genes). As a positive control set for human promoters, we selected +/- 500 bp from the center of the transcription start site of each ZNF target gene and as a negative control set we selected “enhancer regions”, which we define as 1000 nt regions located from −10,000 to −11,000 nt upstream of the promoter of each ZNF gene bound by H3K9me3 at its 3′ end (note that if these enhancer regions overlapped any known promoter regions, they were excluded from analysis). Each of the 3 sets of sequences (upstream negative controls, ZNF promoters, and ZNF 3′ ends) from the 121 ZNF genes were analyzed for the occurrence of the position weight matrix (as defined in [16]) for the promoter-enriched motifs CAAT, CREB, NRF2, GC, Inr, NRF1, Sp1, and YY1 using the program MAST. Motifs with an E-value below 10 were used for evaluation and each region was searched for the motif in the forward and reverse orientation. Then, the number of motifs per 1kb region was determined for each category (upstream negative controls, ZNF promoters, and ZNF 3′ ends) to normalize the analysis (Figure 1A). As expected, most of the promoter motifs are found in the ZNF promoters and some motifs are found in the enhancer regions. However, the 3′ ends of the ZNF genes do not contain the known promoter motifs.

Of course, it is possible that promoters embedded in ZNF 3′ exons might be regulated by a different set of transcription factors and thus would not show enrichment of the common promoter motifs. Therefore, we next investigated whether we could associate the H3K9me3 binding sites with 5′ ends of transcripts. For these analyses, we obtained all CAGE tags available from the Riken CAGE database (http://gerg01.gsc.riken.jp/cage/download/hg17prmtr/chromosomes/). The number of CAGE tags were counted for each region (upstream negative controls, ZNF promoters, and ZNF 3′ ends) and normalized for region size. As shown in Figure 1B, the set of ZNF promoters could be associated with the CAGE tags but very few CAGE tags could be found that corresponded to the upstream enhancer regions or to the H3K9me3 sites at the ZNF 3′ ends. Thus, bioinformatic analyses do not provide strong support for the hypothesis that ZNF 3′ exons are promoter regions.

**Figure 2:**
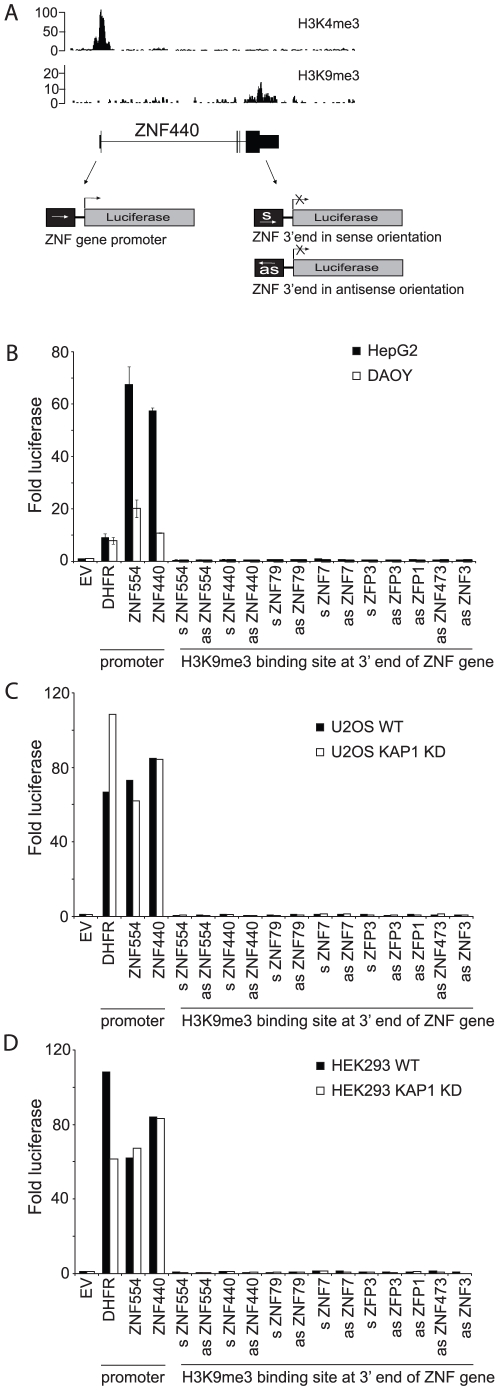
The 3′ ends of ZNF genes do not display promoter activity. A) ChIP-seq signal tracks from Ntera2 cells are shown for the *ZNF440* gene. H3K4me3 binding is observed at the promoter (top panel), while H3K9me3 localizes to the 3′ end of the *ZNF440* gene (bottom panel). The number of sequenced tags is plotted on the y-axis. Shown below the *ZNF440* gene schematic are representative constructs for the experiments shown in panels B–D. Promoter regions (from ∼500 bp upstream to ∼100 bp downstream of TSS) and 3′ regions bound by H3K9me3 in vivo were cloned in front of the luciferase cDNA; the 3′ regions were cloned in either the sense (s) or antisense (as) direction (see Table S2 for coordinates of the genomic fragments used for promoter analyses). B–D) Luciferase assays were performed to test for promoter activity at 3′ ends of ZNF genes. The DHFR, ZNF440 and ZNF554 promoters were used as positive controls and an empty vector (EV) was used as a background control. Promoter activity was tested in Ntera2 and DAOY cells B), in U2OS cells (C) and in HEK293 cells (D). In addition, the U2OS and HEK23 cells were also stably transfected with a KAP1 shRNA construct (indicated as KAP1 KD). Fold luciferase was determined based on the empty vector control and is plotted on the y-axis.

As a third approach to test the hypothesis that the 3′ exons of ZNF genes are promoters, we cloned a set of the 3′ exons into a luciferase reporter vector (Figure 2A). As positive controls for these assays, we used the *DHFR* promoter (a well-studied housekeeping promoter that is active in all cell types) and the promoter regions of the *ZNF554* and *ZNF440* genes. Eight ZNF 3′ ends were analyzed for promoter activity. It was possible that the ZNF 3′ ends could be promoters oriented such that they produced antisense transcripts relative to that same ZNF gene or they could be alternative upstream promoters of a downstream gene. Therefore, we cloned 5 of the ZNF 3′ ends in both orientations upstream of the luciferase cDNA so that we could analyze transcriptional activity in both directions. To begin, HepG2 and DAOY cells were transfected with the various reporter constructs and luciferase activity was determined. As expected, the *DHFR*, *ZNF554*, and *ZNF440* promoters were active in both cell types. However, none of the ZNF 3′ ends showed any promoter activity in either orientation (Figure 2B). We have shown previously that the ZNF 3′ ends bound by H3me3K9 are also bound by KAP1 (also called TRIM28) [10]. KAP1 is a corepressor that recruits the histone methyltransferase SETDB1, resulting in trimethylation of histone H3 on lysine 9 [10], [17]. It is postulated that when KAP1 is located at promoter regions, it can function to repress transcription by recruiting a histone methyltransferase and other repressive proteins such as HP1 family members [18]. Thus, it was possible that potential promoter activity from the ZNF 3′ exons was suppressed due to KAP1-mediated repression. Therefore, we also transfected the various promoter-luciferase constructs into both U2OS cells and HEK293 cells that were stably expressing shRNAs against KAP1 [18], [19]. However, even under conditions of KAP1 knockdown, none of the tested 3′ ends of the ZNF genes functioned as a promoter in the luciferase reporter assays (Figure 2C, 2D). Therefore, neither bioinformatics analyses nor a functional assay provides support for the hypothesis that the 3′ exons of the ZNF genes are cryptic promoters, even though they are bound by a mark (H3K9me3) often found at promoter regions.

**Figure 3:**
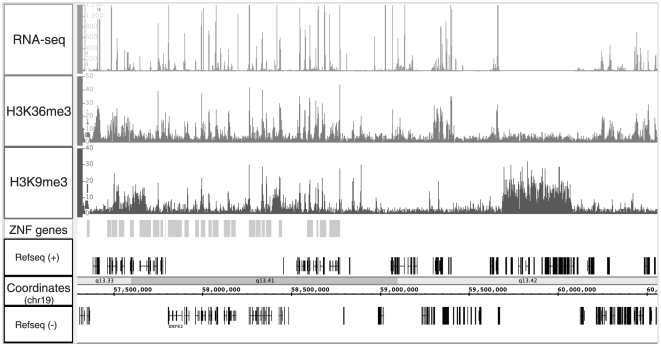
ZNF3′ ends are marked by H3K36me3. Shown for a region of chromosome 19 (hg18 coordinates) are the RNAseq, H3K36me3, and H3K9me3 patterns for hES cells. Also shown is a track that indicates the position of the ZNF genes within that region. The hES RNAseq and ChIP-seq experiments were performed as part of the NIH Roadmap Epigenome Mapping Consortium (http://www.roadmapepigenomics.org/). The RNAseq data and the H3K4me3 and H3K9me3 modifications of a small set of loci have been previously analyzed as part of a previous publication [22].

**Figure 4:**
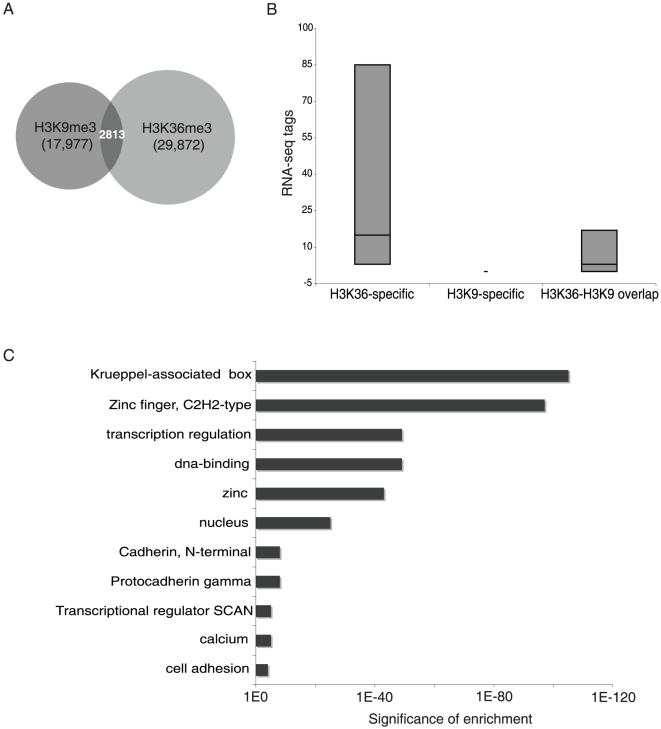
ZNFs are the largest category of genes that have both H3K9me3 and H3K36me3 marks. (A) Shown is a Venn diagram indicating the number of regions bound by H3K9me3, H3K36me3, and both marks, as determined by analysis of hES cell ChIP-seq data using Sole-searchv2. (B) The number of RNA-seq tags corresponding to regions identified as bound by H3K9me3 alone, H3K36me3 alone, and by both H3K9me3 and H3K36me3 is plotted. (C) The regions bound by both H3K36me3 and H3K9me3 were analyzed using the DAVID gene ontology program [21]. The gene nearest to each binding site was chosen for analysis; shown are the enriched terms and P-value of enrichments.

The 3′ exons of ZNF genes have histone modifications characteristic of both silenced and active regions. As indicated above, H3K9me3 is generally considered to be a mark of repression due to its colocalization with the KAP1 corepressor and the HP1 family of silencing proteins. However, the unusual location of the H3K9me3 marks at the 3′ exons of ZNF genes may suggest that this mark is functioning in a different manner for this set of genes. To investigate whether the ZNF genes that are bound by H3K9me3 are active or repressed, we analyzed the ZNF genes for additional histone modifications, including ChIP-seq data for H3K4me3, H3K4me1, H3AcK9, H3K27me3, and H3K36me3. The only other mark that was found at the ZNF 3′ ends was H3K36me3, a mark of transcriptional elongation. As shown in Figure 3, the ZNF 3′ ends that have H3K9me3 also have high levels of the H3K36me3 mark. Although the binding patterns of H3K9me3 and H3K36me3 are similar over many ZNF genes, there are clearly many regions identified by H3K9me3 but not by H3K36me3 (e.g. 59680000 and 60060000 which includes a cluster of killer cell and leukocyte immunoglobulin-like receptors). As expected, there are also many regions identified by H3K36me3 and RNA-seq (e.g. the RPS9 gene at ∼5940000) but not by H3K9me3. To examine the relationship between H3K9me3 and H3K36me3 on a genome-wide scale, we analyzed hES cell ChIP-seq data obtained using the H3K9me3 and H3K36me3 antibodies. To do so, we first needed to modify Sole-search, our ChIP-seq peak-calling program [20]. This program was originally developed to analyze the binding patterns of site-specific DNA binding proteins, which produce very narrow peaks in ChIP-seq analyses. The binding patterns of modified histones, in particular H3K9me3 and H3K36me3, are not narrow but instead can spread over large regions and exhibit very jagged enrichment profiles. Therefore, we modified the program such that it allows “binding regions” rather than narrow peaks to be identified; see Supplementary Information S1 and Figure S1 for more details about Sole-searchv2. A comparison of the number of called peaks and genomic coverage using the standard versus the histone parameters is shown in Table 1; the most important difference is that a larger region of the genome is identified as being covered by histone marks using the modified program. Using the histone-specific program parameters, we identified 20,744 H3K9me3 regions and 32,685 H3K36me3 regions. However, only 2,813 regions are covered by both types of modified histones (Figure 4A). We further characterized the 2,813 regions marked by both H3K9me3 and H3K36me3. First we determined how many RNA-seq tags mapped to the H3K36-specific regions, the H3K9me3-specific regions, and the regions covered by both marks. As expected, the regions covered only by H3K36me3 were transcribed and the regions covered only by H3K9me3 were not transcribed (Figure 4B). Interestingly, the dual bound regions display modest transcription levels, suggesting that the presence of a H3K9me3 mark is not incompatible with transcription. Then, we mapped the dual covered regions to the nearest gene and used the DAVID gene ontology program [21] to identify specific functional classes of genes showing these dual patterns. Strikingly, the main gene category identified was Krueppel-associated C2H2 zinc finger genes (243 genes), with a minor category of cadherins (25 genes) (Figure 4C). The cadherins are all clustered on chromosome 5 and consist of a tandem array of alternatively used 5′ exons and common 3′ exons. Unlike the 3′ exon-specific marking of the ZNFs by H3K9me3 and H3K36me3, it is the promoter regions and alternatively used 5′ exons of the cadherin genes that are dually bound by the two histone marks.

**Table 1:** Analysis of ChIP-seq data.

	Original Sole-Search		Modified Version		
	Peaks	Total coverage (bp)	Peaks	Total coverage (bp)	Fold difference
H3K4me3	9,868	3,416,942	10,165	14,139,035	4.1
H3K36me3	27,811	9,672,419	32,685	65,057,655	6.7
H3K9me3	26,728	9,212,672	20,744	35,215,276	3.8

Shown are the number of peaks called and the total number of bp covered by each peak set for H3K4me3, H3K36me3, and H3K9me3 using the original Sole-search program or the program which has been modified to identify broad regions covered by modified histones. Also shown in the increase in genome coverage (fold difference) that results when using the modified peak calling program. Both the original and the modified program can be accessed at http://chipseq.genomecenter.ucdavis.edu/cgi-bin/chipseq.cgi.

**Figure 5:**
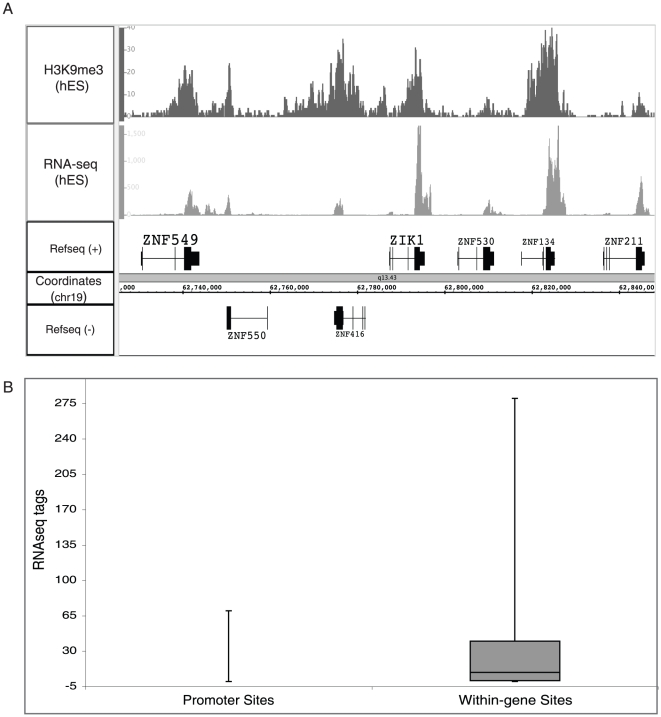
ZNF 3′ exons are transcribed. (A) Shown for a region of chromosome 19 is the ChIP-seq pattern for H3K9me3 and the RNA pattern obtained using RNAseq, both from hES cells. (B) Shown on the Y axis are the number of RNA-seq tags from hES cells corresponding to H3K9me3 bound regions that are classified as promoters (183 sites) or intragenic (433 sites).

The H3K9me3 mark is not repressive when located at ZNF 3′ ends. Our ChIP-seq results suggested the possibility that the ZNF genes may be imprinted, with one allele transcribed (and covered by H3K36me3) and one allele repressed (and covered by H3K9me3). To test the hypothesis that the contradictory modifications are due to imprinting, we examined levels of RNA corresponding to the ZNF genes in hES cells, using RNA-seq data [22]. We found that the ZNF exons that show enrichment for H3K9me3 are transcribed in hES cells (Figure 5A). To further investigate the role of H3K9me3, we identified the top 716 H3K9me3 sites and separated them based on whether they were located in a promoter region (183 sites) or within a gene (433 sites). As shown in Figure 5B, promoter regions bound by H3K9me3 are associated with a very low number of RNA-seq tags in hES cells whereas regions within genes that are bound by H3K9me3 are associated with a larger number of RNA-seq tags. Thus, both RNA analyses and chromatin modification analyses suggest that ZNF genes having H3K9me3 on their 3′ exons are transcribed from at least one allele.

If only one allele of the ZNF genes is transcribed, one might expect that the H3K9me3 and H3K36me3 peak heights at the dually covered regions would be approximately half the peak heights of the marks at the singly covered sites (because only one allele would contribute to each signal in the dually covered sites whereas both alleles would contribute to the signal in the singly covered sites). However, the average height of the H3K9me3 peaks at 3′ ZNF exons that had both marks was higher than the average height of the H3K9me3 peaks at 3′ ZNF exons that had only H3K9me3. Similarly, the average height of the H3K36me3 peaks at 3′ ZNF exons that had both marks was higher than the average height of the H3K36me3 peaks at 3′ ZNF exons that had only H3K36me3 (data not shown). Although not conclusive, these results suggested that the H3K9me3 and H3K36me3 marks on the dually bound ZNF 3′ exons may not be limited to only one allele. We next used the program ssahaSNP to identify single nucleotide polymorphisms (SNPs), as compared to the reference genome sequence, that were located within genes targeted by both H3K9me3 and H3K36me3 on chromosome 19. We then searched for these SNPs within the RNA-seq dataset. Out of 72 SNPs that were identified, 40 SNPs and 3 wt nts were fixed in the RNA sequence data (the SNP or wt nt composed >90% of all nucleotides sequenced for this position). This could suggest either that the ZNFs containing these SNPs were expressed in an allele-specific manner or that the SNP was fixed in the ES cell genome. However, we did identify 29 cases where both wt and SNP alleles are present in the RNA-seq tags approximately equally (Table 2 and Table S3). Since both alleles are transcribed, the hypothesis that transcription is allele-specific is not supported for these ZNF genes bound by both H3K9me3 and H3K36me3.

**Table 2:** Analysis of RNA-seq SNPs.

	Count
WT fixed	3
SNP fixed	40
Heterogeneous	29
Total	72

The program ssahaSNP was used to identify single nucleotide polymorphisms (SNPs), as compared to the reference genome sequence, that were located within genes targeted by both H3K9me3 and H3K36me3 on chromosome 19. Out of 72 SNPs that were identified, 40 SNPs and 3 wt nts were fixed in the RNA sequence data (the SNP or wt nt composed >90% of all nucleotides sequenced for this position) but there were 29 cases where both wt and SNP alleles are present in the RNA-seq tags approximately equally.

**Figure 6:**
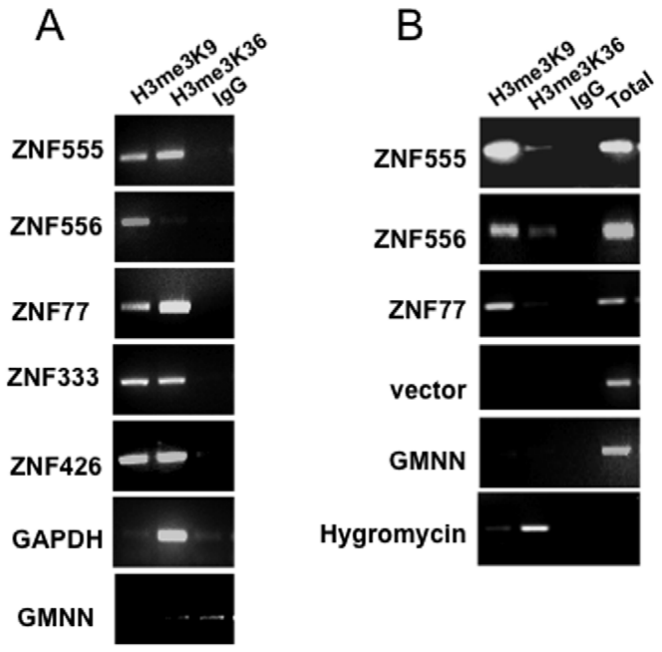
H3K9me3 and H3K36me3 are not co-regulated at ZNF 3′ ends. (A) ChIP experiments were performed in HEK293 cells using antibodies specific for H3me3K9 and H3me3K36; IgG was used as a negative control. Using primers specific for the endogenous chromosomal regions, the 3′ exons of 4 ZNFs shown by ChIP-seq of hES cells to be bound by both H3K36me3 and K3K9me3 (*ZNF555*, *ZNF77*, *ZNF333*, and *ZNF426*) were show to be bound by both marks in the HEK293 cells. In contrast, *ZNF556*, which bound only by H3K9me3 (as was predicted from the ChIP-seq binding patterns from hES cells. The GAPDH gene was used as a positive control for H3K36me3 and a negative control for H3K9me3 and the *GMNN* promoter served as a negative control for both marks. (B) eChIP experiments were performed (using primers specific for the regions cloned into the episomal vectors) in HEK293 cells harboring episomal constructs containing the indicated regions. Antibodies specific for H3me3K9 and H3me3K36 were used and IgG was used as a negative control. Episomal constructs harboring the 3′ ends of *ZNF555*, *ZNF556*, and *ZNF77* were analyzed; an empty episomal vector and an episomal vector harboring the *GMNN* promoter were used as negative controls. The hygromycin cDNA (located on the opposite side of the episome from the cloning site) was used as a positive control for H3K36me3.

H3K9me3 and H3K36me3 are not co-regulated. The studies presented above suggest that the H3K9me3 and H3K36me3 marks on the dually bound ZNF3′ exons are not mutually exclusive. We next wished to investigate whether the dual H3K9me3 and H3K36me3 marks are co-regulated. To investigate this hypothesis, we used a method that we have previously developed called eChIP, which allows the in vivo study of a chromosomal region removed from its normal chromosomal location [23]. We cloned the 3′ ends of ZNF555, ZNF556, and ZNF77 into the episomal vector, introduced this vector into HEK293 cells that express EBNA1 (required for the maintenance of the episome), and then performed a ChIP analysis using H3K9me3 and H3K36me3 antibodies. As a control, we first demonstrated that in HEK293 cells the 3′ ends of the endogenous *ZNF555*, *ZNF77*, *ZNF333*, and *ZNF426* genes are bound by both H3K9me3 and H3K36me3 and that, as predicted from the hES ChIP-seq results, the 3′end of *ZNF556* is bound only by H3K9me3 and not by H3K36me3 (Figure 6A). We also showed that the *GAPDH* gene is bound only by H3K36me3 and not by H3K9me3, as expected for a highly transcribed gene and that the *GMNN* promoter is not bound by either mark. We next analyzed the episomal constructs. We observed high levels of H3K9me3 on the episomal ZNF 3′ ends, but not on the GMNN promoter, an episomal vector that does not contain an insert, or the hygromycin cDNA region (Figure 6B). In contrast, analysis of the episomal ZNF 3′ ends showed that they are not marked by H3K36me3. As a control to demonstrate that the H3K36me3 antibody could detect the H3K36me3 mark on an episome, we analyzed the hygromycin cDNA that is located on the same episomal vector as the GMNN promoter and cloned downstream of a eukaryotic promoter. Expression of hygromycin is used to select the stable episomes and thus this cDNA is transcribed and we could detect H3K36me3 in this region of the episome. These results demonstrate that although both H3K9me3 and H3K36me3 marks show very high enrichment at the 3′ ends of ZNFs, these two marks are not co-regulated. H3K36me3 is produced by SETD2 [24] and is thought to be recruited to the transcriptional elongation complex via interaction with Ser 2-phosphorylated RNA Polymerase II (reviewed in [25]); the levels of Ser 2-phosphorylated RNA polymerase II, and thus also the levels of H3K36me3, increase from the 5′ to 3′ direction throughout the transcription unit. Our results show that the H3K9me3 deposition is not dependent on the same mechanism as H3K36me3 deposition since the episomes have the H3K9me3 but not the H3K36me3 mark. In fact, the high H3K9me3 mark on the episomal ZNF 3′ exons indicates that the cis elements required for the mark are all present on the episomes. We have recently shown that the KAP1/SETDB1 histone methylation complex colocalizes with H3K9me3 [26]. Thus, the episomes must contain the cis elements that can recruit the KAP1/SETDB1 complex.

**Figure 7:**
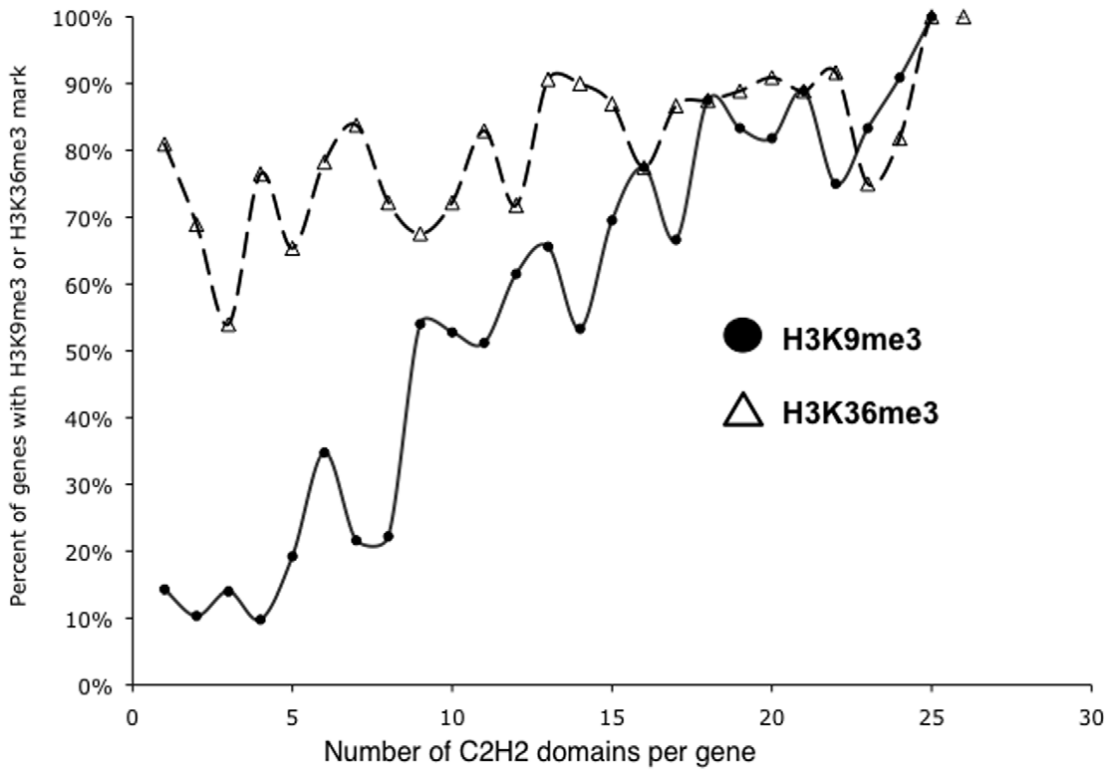
H3K9me3 enrichment at ZNF 3′ ends correlates with the number of finger domains. Shown is the percentage of ZNF genes having the specified numbers of zinc fingers that are targeted by H3K9me3 or H3K36me3 (ZNF genes having from 1 to 25 zinc fingers were used for this analysis).

H3K9me3 at ZNF 3′ exons corresponds with the number of tandemly repeated finger domains. ZNF genes contain multiple, highly conserved finger domains in their 3′ exons. In essence, these finger domains create highly repetitive genomic regions. H3K9me3 has been shown to bind to other repetitive elements, such as transposons and centromeric repeats [13], [14], [15]. If the H3K9me3 was binding to the ZFN 3′ ends simply as a consequence of the repetitive nature of these genomic regions, then we would expect the likelihood of H3K9me3 to be bound to a ZNF gene to increase as the number of finger domains increases. To test this possibility, we classified the ZNF genes into sets depending on how many tandomly repeated finger domains were present in the gene and then plotted the percentage of each set that is covered by H3K9me3 (Figure 7). We note that ZNFs that have very few finger domains are rarely covered by H3K9me3, whereas ZNFs having greater than 15 domains are almost always covered by H3K9me3. Clearly, there is a strong relationship between the number of tandemly repeated finger domains and the likelihood of being covered by H3K9me3. In contrast, there is no relationship between the number of repeated finger domains and the likelihood of being covered by H3K36me3. As a further analysis of the repetitive nature of H3K9me3 vs. H3K36me3 peaks, we examined 20,744 H3K9me3 peaks and 20,744 H3K36me3 peaks and searched for tandem repeats using the program XSTREAM, identifying sequences that are 50bp or larger, repeated at least 5 times in the human genome, and having at least 60% conservation between repeat elements. We then calculated the percentage of each peak that was a repetitive element. We found that more of the H3K9me3 peaks consisted of a high percentage of repetitive regions. For example, there are ∼17 times more H3K9me3 peaks that consist of 91-100% repetitive elements (see Figure S2).

## Discussion

The focus of this study was to characterize the contradictory chromatin marks found at the 3′ ends of hundreds of ZNF genes in the human genome. Because H3K9me3 has been shown to be promoter-localized [9], [27], [28], we first investigated the possibility that the 3′ ends of ZNF genes harbor cryptic or previously uncharacterized promoters. However, neither bioinformatic nor experimental approaches support the hypothesis that the 3′ exons of ZNFs are promoter regions. We next analyzed genome-wide ChIP-seq data for H3K9me3 and H3K36me3 and found that although there are ∼18,000 H3K9me3 and ∼30,000 H3K36me3 regions in human ES cells, these two modified histones are found together at very few genomic locations. The reason for the low overlap is that H3K36me3 is a mark of transcriptional elongation (and thus covers intragenic regions downstream of promoters) whereas H3K9me3 is mainly found at promoter regions or in large transcriptionally inactive domains. In fact, ZNF 3′ exons constitute the vast majority of the overlapping H3K9me3 and H3K36me3 sites in the human genome. Although the patterns of H3K9me3 and H3K36me3 are very similar on ZNF genes, we have used episomal ChIP assays to show that the marks are not co-regulated.

**Figure 8:**
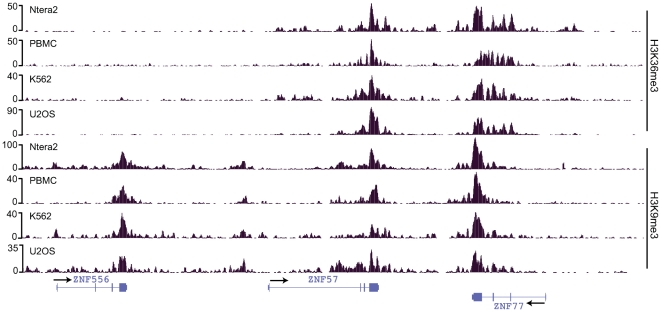
ZNF 3′ exons are covered by H3K9me3 and H3K36me3 in both pluripotent and differentiated cell types. Shown are the ChIP-seq profiles of H3K9me3 and H3K36me3 for a section of chromosome 19 from Ntera2 (pluripotent testicular embryonal carcinoma from a 22 year old male), Proliferating Blood Mononuclear Cells (PBMC) from a 28 year old male), K562 (chronic myelogenous leukemia from a 53-year-old female), and U2OS (moderately differentiated sarcoma of the tibia of a 15 year old girl) cells. The number of sequenced tags is plotted on the y-axis, the positions of 3 ZNF genes are indicated on the x axis (the direction of transcription is indicated by the arrows), and the antibody used for each experiment is indicated on the right side of the figure.

It is thought that the H3K9me3 histone modification serves as a transcriptionally repressive mark. However, others have shown that H3K9me3 can be found to co-localize with RNAPII or H3K9Ac on promoters [9], [27] and is also present on some transcribed regions [28], [29]. Therefore, simply identifying a region as being marked by H3K9me3 does not provide information as to whether the gene is transcribed. There are other examples of contradictory marks being located on certain genes. For example, imprinted genes have both H3K9me3 and H3K4me3 marks, with the repressive H3K9me3 being located on one parental allele and the active H3K4me3 mark being enriched on the other parental allele [30]. For these cases, the contradictory marks are located at the promoter regions and only one allele is transcribed. Another set of contradictory chromatin marks includes the bivalent promoters that have both H3K4me3 and H3K27me3 [31]. In this case, the repressive H3K27me3 mark is dominant and the genes are not transcribed. Our analyses show that the ZNF genes that have both H3K9me3 and H3K36me3 are transcribed (unlike the bivalent marks) and, for at least some ZNFs, the transcripts are not allele-specific (unlike the imprinted genes). We also note that the nucleosomes at the 3′ ends of the ZNF genes that have the H3K9me3 and H3K36me3 marks do not have either H3K4me3 or H3K27me3 (Figure 2 and data not shown). Another distinction between the dual H3K9me3 and H3K36me3 marks found on ZNF genes and the bivalent H3K4me3 and H3K27me3 marks found on HOX genes is that the H3K4me3 and H3K27me3 bivalent marks are found only on chromatin from embryonic cells whereas the dual H3K9me3 and H3K36me3 marks at 3′ ZNF exons are found in every cell type that we have examined, including both embryonic and adult cells as well as normal and cancer cells (Figure 8).

Because the ZNF genes covered by H3K9me3 at their 3′ exons are transcribed, we suggest that H3K9me3 may not be involved in repressing transcription of these genes. Rather, H3K9me3 may serve to create a more condensed chromatin structure that can inhibit inappropriate homologous recombination between different highly related ZNF genes. We have shown that there is a strong relationship between the number of tandemly repeated finger domains and the likelihood of being covered by H3K9me3 (but not by H3K36me3, which correlates with the transcript level and not the number of fingers in a particular ZNF gene). Also, we have found that when ZNF genes are introduced into cultured human cells, they very often become truncated at their 3′ ends (Iyengar and Farnham, unpublished data). It is possible that the “unprotected” plasmid DNA corresponding to the ZNF 3′ exons serves as a recombination template, resulting in recombination and mutation of the introduced gene. Future studies are required to address the possible role of H3K9me3 (and the enzyme complexes that mediate this histone modification) in suppression of homologous recombination amongst the hundreds of ZNF genes.

Finally, we note that to accurately compare genomic regions enriched for spreading histone modifications such as H3K9me3 and H3K36me3, we developed a modified peak calling program that takes into account duplicated regions of the genome, biases in sequencing of certain regions, and the jagged profile of regions enriched for histone modifications. This program, Sole-searchv2, is publicly available at http://chipseq.genomecenter.ucdavis.edu/cgi-bin/chipseq.cgi.

## Methods

Luciferase assays: Luciferase assay constructs were created by ligating PCR products into pGL4-11 (Promega) upstream of the luciferase gene. The pGL4-11b-DHFR control plasmid was provided by SwitchGear Genomics. Plasmids containing *ZNF440* or *ZNF544* gene promoters and 3′ regions of ZNF genes were cloned using XhoI/HindIII restriction sites. All other ZNF constructs were created by blunt cloning into SmaI restriction site. For luciferase assays, HepG2 (ATCC #HB-8065), DAOY (ATCC #HTB-186), U2OS(ATCC #HTB-96)**,** and HEK293 (ATCC #CRL-1573**)** cells were grown in DMEM supplemented with 10%FBS, 2 mM L-Glutamine, 100 U/mL Penicillin/Streptomycin. Stable KAP1 knockdown cell lines K928-cI10 (HEK293) and U2OS-K4 (U2OS) were kindly provided by David Schultz [18], [19]. K928-cl10 cells were grown as above with the addition of 10 µg/ml puromycin, U2OS-K4 cells were grown in the presence of 200 µg/ml zeocin. Cells were plated in 24 well dishes at a density of 30,000 cells per well. The following day, cells were co-transfected with 100 ng luciferase plasmid and 5 ng Renilla reporter plasmid phRL-SV40 (Promega) using Effectene (QIAGEN) according to the manufacturer's instructions. Cells were lysed 48 hours post transfection and luciferase activity was determined using the Dual-Luciferase Reporter Assay System (Promega) according to the manufacturer′s protocol. Luciferase values were normalized using Renilla values.

ChIP-seq. ChIP-seq was performed as described in Supplementary Information S1. Briefly, cells were crosslinked, chromatin was extracted and sonicated to an average size of 300–500 bp, and individual ChIP assays were performed using antibodies to modified histones and protein G-coupled magnetic beads. ChIP libraries were created as described previously [32], using 15–18 cycles of amplification. Libraries were run on a 2% agarose for gel purification. Library DNA was quantitated using either a Nanodrop or a BioAnalyzer and sequenced on an Illumina GA2 by the DNA Technologies Core Facility at the University of California-Davis (http://genomecenter.ucdavis.edu/dna_technologies/) or at the University of British Columbia Genome Science Center. Lists of all the ChIP-seq experiments and information concerning the antibodies used for each histone mark are provided in Table S1 and the coordinates of the primers used for ChIP assays are listed in Table S2; see Supplementary Information S1 for antibody validation. The hES ChIP-seq experiments were performed as part of the NIH Roadmap Epigenome Mapping Consortium (http://www.roadmapepigenomics.org/) and a small set of loci have been previously analyzed using these datasets [22].

ChIP-seq peakcalling. Peaks were identified using version 2 of the Sole-search software. Version 2 of this program has been designed to run more quickly and efficiently on the increasingly large ChIP-seq data sets, to more accurately analyze ChIP-seq data from any species, and to improve peak-calling for proteins and histone modifications which are characterized by spreading. Several changes, as compared to the original Sole-search program [20], were implemented to achieve these goals. This new software is comparable to its predecessor in the ability to accurately determine transcription factor binding sites but greatly improves peak-calling for spreading marks, such as histone modifications; see Supplementary Information S1 and Figure S1 for more details.

Episomal ChIPs (eChIPs): All regions to be cloned into the episomal vector (with the exception of *GMNN* which has been described previously in [23] were amplified from Ntera2 genomic DNA by PCR with *Pfx Polymerase* (Invitrogen) using primers that introduced unique XhoI and HindIII sites immediately 5′ and 3′ to the region of interest, respectively. The target sequences were then cloned into the pFC62 episome using the XhoI and HindIII sites; successful cloning of the various inserts was confirmed by sequencing at the UC Davis Sequencing Facility. The episomal constructs (1 µg) were transfected into HEK293c18 cells stably expressing EBNA-1 in 6-well dishes using FuGene 6 Transfection Reagent (Roche) according to manufacturer's recommendations. Forty-eight hours after transfection, cells were selected in medium containing 500 µg/ml of G418 (Sigma) and 200 ug/ml Hygromycin B (CellGro), and drug resistant colonies were pooled and expanded. Pooled colonies were assayed for the presence of the correctly sized episomal insert using primers that flanked the cloning site and genomic DNA prepared from the stably transfected cells. To perform the eChIP assays, HEK293cl18 cells stably transfected with a particular episome were harvested when the cells were ∼75% confluent. Cells were cross-linked and chromatin was prepared and analyzed using our standard ChIP protocol (http://www.genomecenter.ucdavis.edu/farnham) with a few modifications. Namely, ∼20 ug of chromatin was used for each IP and the amount of antibody was greatly reduced (0.2 ug of Anti-H3me3K9 (Abcam, #8898), Anti-H3me3K36 (Diagenode, # pAb-058-050), or non-specific rabbit IgG antibody (Alpha Diagnostics, # 20009-5) was used). Complexes were recovered with 10 uls of StaphA cells for 15 minutes at room temperature. Washes, elution and purification of the ChIP samples were performed according to the standard protocol. PCR reactions were performed using only 1 ul of the immunoprecipitated sample and amplified for only 28 cycles.

## Supporting Information

Supplementary Information S1 This file includes a description of Sole-search version 2, antibody validation documents, and the ChIP-seq protocol.
(PDF)

Figure S1 **Major steps implemented in Sole-searchv2.** The original Sole-search program has been modified to improve the ability to determine statistically and biologically significant peaks in both transcription factor and histone modification datasets. (A) Input data is smoothed and duplication and deletion events are determined based on fold coverage of these regions compared to average coverage. Next, specific regions of the genome that have a higher sequence coverage than expected by chance, due to experimental method, are determined using a t-statistic. Raw ChIP-seq data is normalized based on duplication event copy number and enrichment of input reads, so that the data to be analyzed will reflect a single copy genome without sequencing bias (see the track corresponding to “Normalized ChIP-seq data”). (B) The second, optional step smoothes data that spreads over large regions (e.g. data from histone modification ChIP-seq experiments), using a sliding average, so that non-uniform “mountain range” peaks are more easily detectable as broad regions. Previously, these regions would be identified as having many, distinct peaks. Also certain smaller peaks would fail to be detected. This smoothing step allows detection of both broad regions and smaller. (C) The third major step determines a statistically significant peak height cutoff. Tags are randomly sampled from the input dataset to create bins. Tags are then shuffled within the bins. Height cutoff is determined based on a user-defined FDR. The cutoff value increases until the number of peaks found within the randomly generated background is sufficiently low, compared to the number of peaks found in the ChIP-seq dataset at the same height.
(PDF)

Figure S2 **20744 H3K9me3 peaks and 20744 H3K36me3 peaks were searched for tandem repeats using the program XSTREAM (** http://www.ncbi.nlm.nih.gov/pmc/articles/PMC2233649/ **), identifying sequences that are 50bp or larger, repeated at least 5 times in the human genome, with at least 60% conservation between repeat elements.** The percent of each peak that was a repetitive element was then calculated. A greater number of the H3K9me3 peaks had high percentages of repetitive regions. For example, there are ∼17 times more H3K9me3 peaks that consist of 91-100% repetitive elements.
(PDF)

Table S1 **Summary table of ChIP-seq data sets.**
(PDF)

Table S2 **List of primers used in this study.**
(PDF)

Table S3 **RNA-seq SNP frequencies.**
(XLSX)
