# Supplementary material for: Characterization of the Contradictory Chromatin Signatures at the 3′ Exons of Zinc Finger Genes
Source: PLoS One. 2011 Feb 15;6(2):e17121. doi: 10.1371/journal.pone.0017121 (PMC3039671; doi:10.1371/journal.pone.0017121)
Supplement: Supplementary Information S1 — This file includes a description of Sole-search version 2, antibody validation documents, and the ChIP-seq protocol. (PDF) [file pone.0017121.s001.pdf]

Blahnik_Supplementary Information

Solesearchv2

Version 2 of the Sole-search software has been designed to run more quickly and efficiently on the increasingly large ChIP-seq data sets, more accurately analyze ChIP-seq data from any species, and improve peak-calling for proteins and histone modifications which are characterized by spreading. Several changes, as compared to the original Sole-search program (Blahnik et al. 2010), were implemented to achieve these goals. This new software is comparable to its predecessor in the ability to accurately determine transcription factor binding sites but greatly improves peak-calling for spreading marks, such as histone modifications.

The first step of the program begins by determining regions in the input genome that are either duplicated or deleted when compared to the reference genome and then estimates copy number of the duplicated regions. Furthermore, this step now determines distinct regions of the Input dataset that are sequenced more often than expected by chance, due to the experimental method of library making and sequencing. To do this, input data is first normalized for duplication events then is binned using a sliding window of 30bp. This data is then smeared using a sliding average, where the center bin of a 1020bp window receives the average value of all of the bins in the window. Bins with more sequence data than would be obtained by chance are then determined using a t-statistic. For this step, the user defines the alpha value. A low alpha value, such as 0.0001, will determine regions of the genome that are most significantly enriched in the sequenced input sample, while a high alpha value, such as 0.1 will identify more (but at a lower level) over-represented genomic regions enriched in the sequenced input. Finally, the ChIP-seq data, binned into 30bp bins, is normalized based on both the copy number of duplicated regions and on the enrichment values of regions determined to be over-represented in the sequenced input.

The second step of the Sole-search software is optional and should be implemented when analyzing data that is characterized by spreading, such as H3K27me3, H3K36me3, H3K4me1, and H3K9me3 histone modifications. ChIP-seq datasets for these histone modifications have a non-uniform profile. Since this data does not form distinct “peaks”, but rather has “jagged mountain ranges”, traditional peak-calling programs often fail to capture the entire region covered by the modification. To improve peak calling for these regions, we first smooth the data so that “peaks” are more uniform. This is done using a sliding average; the center bin is assigned the average value of all the bins of a 1200bp window. The same process is repeated for the randomly generated background models.

The third step of the software is very similar to step 2 of the original Sole-search program. Background models are created by randomly sampling reads from input data, determining how many bins would contain sequence data, based on the number of tags sequenced in the ChIP-seq experiment, and scrambling the reads within these bins, as explained in Blahnik et al, 2010. Peak height cutoff is determined by a user defined false discovery rate (FDR). Peak height cutoff value is increased until the number of peaks in the randomly generated background files is sufficiently low, as compared to the peak count in the ChIP-seq dataset. We suggest starting with an alpha value of 0.0001 and an FDR of 0.01 for histone modification data, and an alpha value of 0.001 and an FDR of 0.001 for transcription factors.

Finally, an option was added to merge peaks that are a user-defined distance apart. This option is recommended for use when analyzing histone modification data.

Antibody validation for Blahnik et al.

The antibodies that we used for each experiment are listed in Table S1. Specifically, we used the H3K9me3 antibody from Cell Signalling (catalog number 9754), the H3K9me3 antibody from Abcam (catalog number 8898), the H3K36me3 antibody from Abcam (catalog number 9050), and the H3K4me3 antibody from Cell Signaling Technology (catalog number 9751). All of these antibodies have been used extensively by the ENCODE Consortium and by the Roadmap Epigenome Mapping Centers. They are all very specific and do not crossreact with other modified histones. The specificity of each antibody is documented in the following pages.

H3me3K4 Antibody: **CST Cat#: 9751**

H3K4me3 antibody validation performed by CST
CST Cat#: 9751 Lot#1,2

http://www.cellsignal.com/products/9751.html

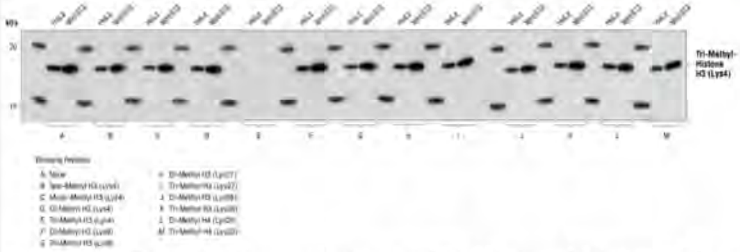

Fig1. Antibody specificity was determined by Western blotting. HeLa and NIH/3T3 cell lysates were probed with Tri-Methyl Histone H3 (Lys4) (C42D8) Rabbit mAb (Panel A) or Tri-Methyl Histone H3 (Lys4) Rabbit mAb pre-adsorbed with 1.5 µM of various competitor peptides (Panels B-M). As shown, only the tri-methyl histone H3 (Lys4) peptide competed away binding of the antibody.

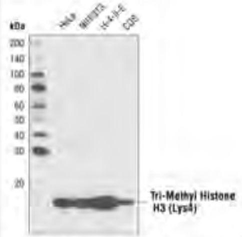

Fig2. Western blot analysis of various cell types using Tri-Methyl Histone H3 (Lys4) (C42D8) Rabbit mAb.

This antibody was also validated for the Farnham lab by dot blot by Bing Ren's lab as part of the REMC Consortium.

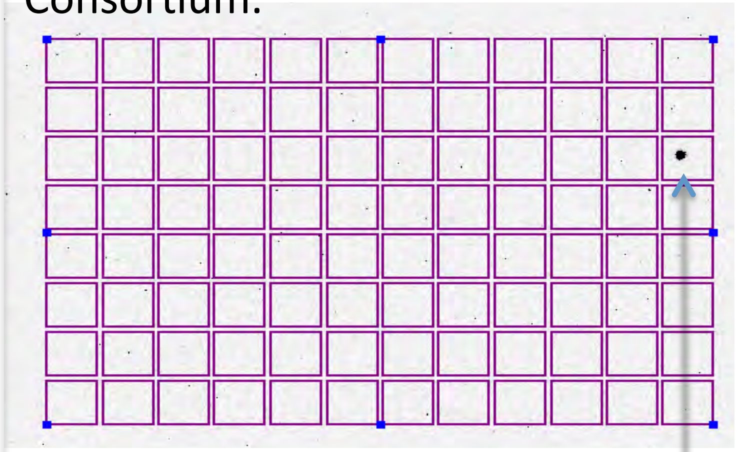

1 = H3K4Me3 (100%)

H3K9me3Cell Signaling Technology 9754

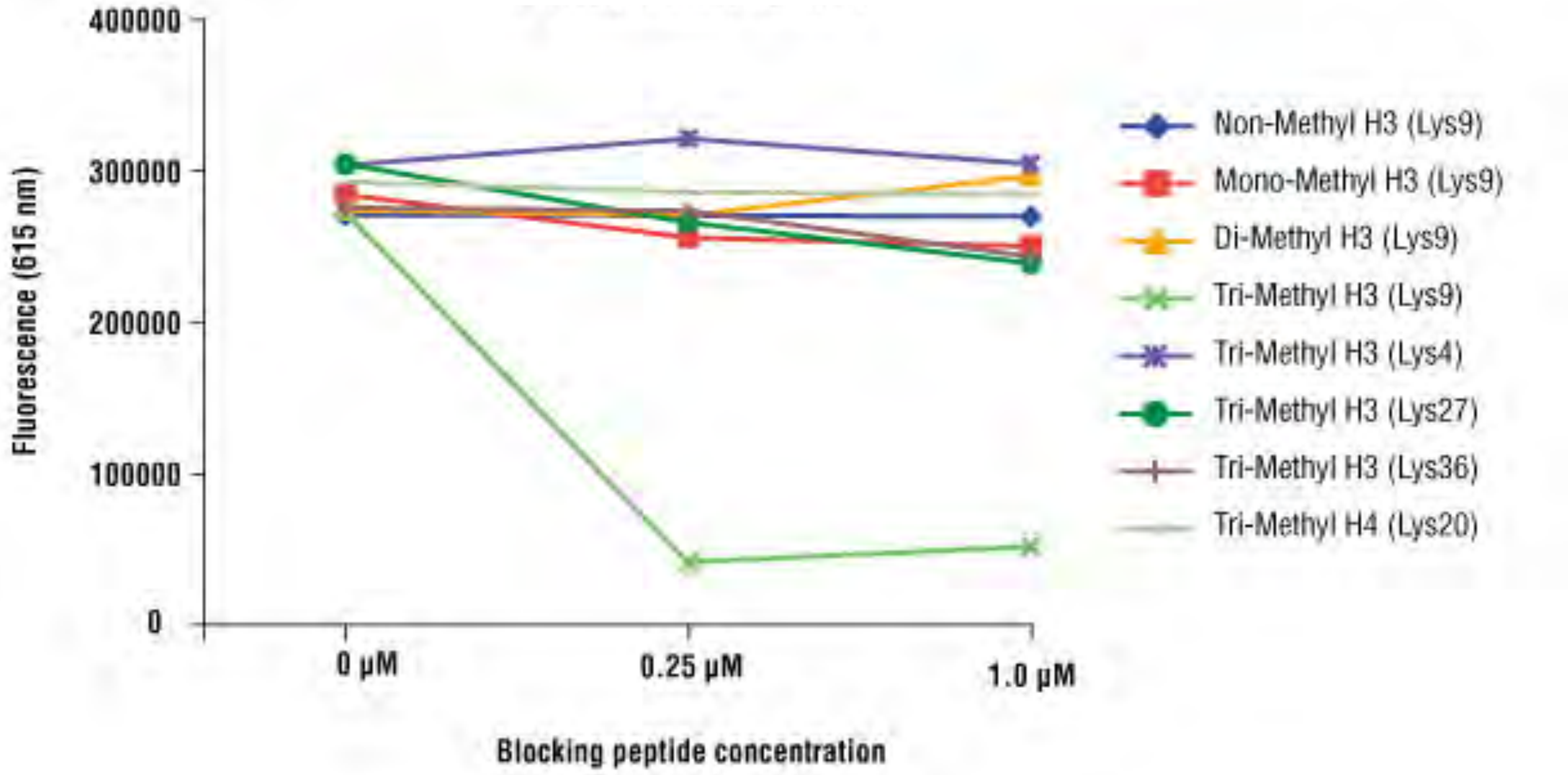

Tri-Methyl-Histone H3 (Lys9) Antibody specificity was determined by peptide ELISA. The graph depicts the binding of the antibody to pre-coated tri-methyl histone H3 (Lys9) peptide in the presence of increasing concentrations of various competitor peptides. As shown, only the tri-methyl histone H3 (Lys9) peptide competed away binding of the antibody. For more details, see http://www.cellsignal.com/products/9754.html

H3K9me3 Abcam, 8898: see validation at http://www.abcam.com/Histone-H3-tri-methyl-K9-antibody-ChIP-Grade-ab8898.html#ab8898_8b.jpg

- Also, this antibody was validated by the Brad Bernstein group as part of the ENCODE Consortium. See below for validation performed at the Broad Institute

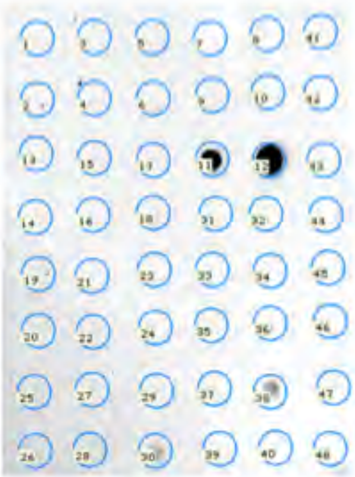

Gel Setup

	1	2	3	4	5	6
A	K4m1 0.01ug	K4m2 0.01ug	K4m3 0.01ug	K9m1 0.01ug	K9m1 0.1ug	Hela 0.01ug
B	K4m1 0.1ug	K4m2 0.1ug	K4m3 0.1ug	K9m2 0.01ug	K9m2 0.1ug	Hela 0.1ug
C	K36m1 0.01ug	K36m2 0.01ug	K36m3 0.01ug	K9m3 0.01ug	K9m3 0.1ug	H3 0.01ug
D	K36m1 0.1ug	K36m2 0.1ug	K36m3 0.1ug	K9ace 0.01ug	K9ace 0.1ug	H3 0.1ug
E	K79m1 0.01ug	K79m2 0.01ug	K79m3 0.01ug	K27m1 0.01ug	K27m1 0.1ug	H4 0.01ug
F	K79m1 0.1ug	K79m2 0.1ug	K79m3 0.1ug	K27m2 0.01ug	K27m2 0.1ug	H4 0.1ug
G	K20m1 0.01ug	K20m2 0.01ug	K20m3 0.01ug	K27m3 0.01ug	K27m3 0.1ug	TBS
H	K20m1 0.1ug	K20m2 0.1ug	K20m3 0.1ug	K27ace 0.01ug	K27ace 0.1ug	TBS

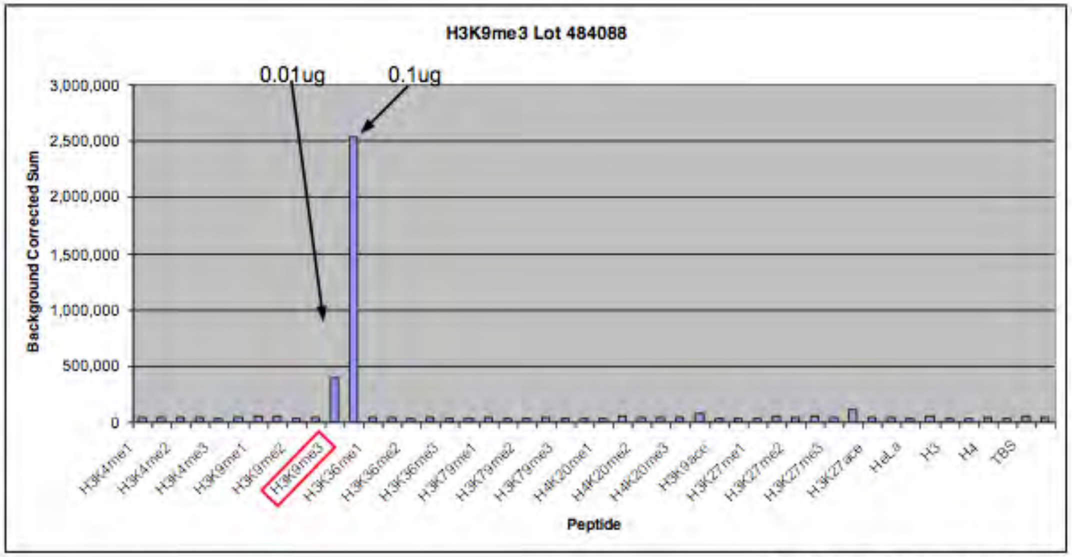

Performed at Broad Institute

Test Date 4/14/2010
User MC

Primary Antibody H3K9me3
Vendor Abcam
Part # ab8898
Lot # 484088
Concentration 1 :2500
Incubation Time 60m

Secondary Antibody
Vendor USB #72560
Part # 72560
Lot # 123941
Concentration 1 :10,000
Incubation Time 30m

Develop Time 5m

Protocol
- Add each peptide into BioDot at above concentrations in 50uL total volume
- Let sit on rocker 60 minutes
- Pull through with vacuum, wash once with 100uL TBS and twice with 200uL TBSTw
- Remove membrane from BioDot and transfer to 10mL Pierce SuperBlock in tray
- Place on rocker for 30 minutes
- Dump off SuperBlock and add Primary Antibody in 10mL total volume
- Place on rocker for 30 minutes
- Wash 3 x 5 minutes in TBSTw
- Add secondary antibody diluted in SuperBlock in 10mL total volume
- Place on rocker for 30 minutes
- Wash 3 x 15 minutes in TBSTw, 1 x 10 minutes in TBS
- Develop in Fluorchem

This antibody was validated by dot blot by Bing Ren's lab as part of the REMC Consortium.

UCD's H3me3K36 Antibody:

Abcam Cat#: ab9050 Lot#395432

1 = H3K36Me3 (100%)

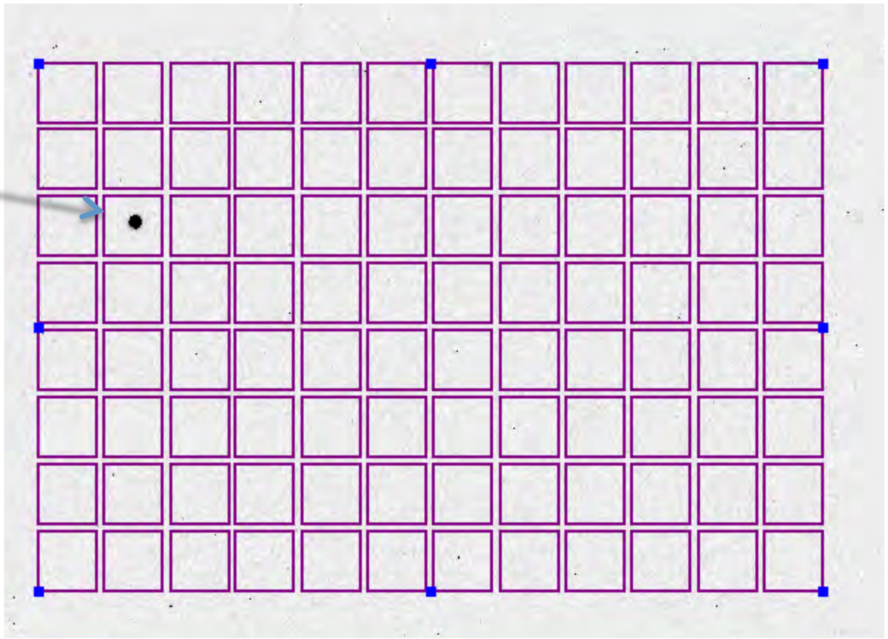

Farnham Lab REMC ChIP and Library Construction Protocol

Preparation of cross-linked cells (Record cell/patient information, date of culture/collection, etc. for future reference)

1. The amount of cells needed for ChIP-seq will vary depending on the histone mark to be analyzed. In general between 1 and 5 ug are used per histone antibody. A minimum of 20 ug is required to analyze all 6 histone marks; this corresponds to 0.5 to 2 x 10^7 cells, depending on the cell type. For primary cells or tissues, the samples can be used immediately or snap frozen in liquid nitrogen and crosslinked at a later date. If using immediately (or after culturing for a few passages), in a chemical hood add formaldehyde (from the 37% stock bottle) directly to the collection media or culture media to a final concentration of 1%. If using frozen cells, in a chemical hood prepare a 1% formaldehyde solution in PBS and add directly to frozen cell pellet; resuspend the cell pellet by pipetting up and down.
2. Rotate the cells in a tightly closed tube (for adherent cells use a shaking platform) for 10 minutes at room temperature. Do not crosslink for longer periods since this may cause cells to form aggregates that do not sonicate efficiently.
3. Stop cross-linking reaction by adding glycine to a final concentration of 0.125 M. We use a 10X (1.25 M) stock solution. Continue to rock or stir at room temp for 5 minutes.
4. For cells crosslinked in suspension, centrifuge cells at 430 rcf for 5 min at 4ºC, discard solution, wash pellet twice with ice-cold 1xPBS (mix by pipetting, pellet cells by centrifugation at 430 rcf for 5 min at 4ºC and discard wash solution). For adherent cells, pour off media and rinse plates twice with ice-cold 1x PBS and pour off wash solution. Using a silicon stopper cut in half, scrape cells in residual PBS and transfer cells from the culture dish to a 15 ml conical tube on ice. Centrifuge the crosslinked cells at 430 rcf for 5 min at 4ºC. It is important to carefully aspirate supernatants so as to not lose cells. Note: media containing formaldehyde should be treated as hazardous waste.
5. Cells may now be used immediately for a chromatin preparation or snap frozen in liquid nitrogen and stored at -80°C, then shipped on dry ice to the Farnham lab.

Preparation of chromatin

Sonication conditions should be optimized for each cell type and chromatin size determined before processing large quantities of cells!

1. If using frozen crosslinked cells, thaw them on ice; keep all cells and chromatin samples on ice at all times. Prepare the cell lysis buffer (1 ml cell lysis buffer per 1x10^7 cells): add Igepal (10 µl per ml cell lysis buffer, agitate at 37°C to dissolve, cool on ice), then add

protease inhibitors [PMSF (10 µl/ml), aprotinin (1 µl/ml) and leupeptin (1 µl/ml)]. Resuspend cell pellet in freshly prepared cell lysis buffer by pipetting. The final volume of cell lysis buffer should be sufficient so that there are no clumps of cells. Incubate on ice for 15 minutes.

2. Homogenize cells using a glass dounce homogenizer (type B) to break open the cells and release nuclei. Homogenize cells on ice with 20 strokes. Omit this step when processing less than 1 million cells.
3. Centrifuge the crosslinked cells at 1,500 rpm for 5 min at 4°C.
4. Discard supernatant and resuspend pellet in nuclei lysis buffer (20 µl of nuclei lysis per 1x10^6 cells; be careful not to use too much NL buffer as it may lead to dilute chromatin) plus protease inhibitors. Incubate on ice for 30 minutes. An optional flash-freezing step may help break open nuclei more efficiently. This step is critical if the homogenizing in step 2 is omitted. Flash freeze nuclei after 30 minutes of incubation in NL buffer, thaw at room temperature. Once thawed, immediately transfer to ice (do not allow samples to warm up to room temperature) and proceed to sonication.
5. Sonicate cells in a cold room and/or on ice to achieve an average chromatin length of 300-500 bp. Wear hearing protection! We use the BioRuptor on high setting for sonication. Volumes between 0.5 ml and 2 ml are sonicated in 15 ml tubes, volumes between 0.1 and 0.3 ml are sonicated in 1.5 ml Eppendorf tubes. The pulse duration, intensity, and number will vary depending on the sonicator, the extent of cross-linking, and cell type. Ideally the least amount of input energy that gives satisfactory fragmentation should be used. We commonly sonicate 30 minutes (pulses of 30 seconds at setting high, 1.5 minutes pause in between pulses). These settings have been used for hES cells and breast cells; T-cells typically need an extra 5-10 minutes of sonication to achieve the desirable size range.
6. Transfer sonicated samples into an Eppendorf tube and centrifuge at 14,000 rpm for 10 minutes at 4°C. Carefully transfer the supernatant (sonicated chromatin) to a new tube while avoiding cell debris. Keep sonicated chromatin at 4°C while performing quantification and determining chromatin size.

Determining chromatin size and chromatin quantification

1. Take an aliquot of your chromatin sample from step 6 above (typically we use chromatin from 1-2 x 10^5 cells).
2. Add ChIP elution buffer to a total volume of 100 µl, add 12 µl 5M NaCl, boil samples in water bath for 20 minutes to reverse cross-links.
3. Allow sample to cool down, add 1 µl DNase-free RNase, incubate 20 minutes at 37ºC. This step is important because the presence of RNA results in false estimation of chromatin size.

4. Purify DNA using a PCR purification kit, elute in 30 µl water. Measure chromatin concentration by NanoDrop and calculate the chromatin yield.
5. Run 1.5-2 µg of chromatin on a 1.2% agarose gel to visualize average size of chromatin. If the chromatin is larger than ~600 bp, repeat steps 1-5 and adjust the sonication conditions (e.g add more pulses).

Chromatin Immunoprecipitation

[For best results, proceed to ChIP assays on the same day as chromatin is prepared]

1. Measure volume of chromatin and divide chromatin as needed. The amount of chromatin needed for each ChIP reaction varies depending on the histone modification. For histone marks covering a small portion of the genome displaying sharp peaks, such as H3K4me3, we typically use 1-2 µg of chromatin. For spreading histone marks such as H3K9me3 or H3K36me3 that cover large portions of the genome we prefer to use 5 µg chromatin. (Optional: Include an IgG negative control sample along with your experimental antibodies. However, oftentimes no chromatin can be spared for a control ChIP. One can use the activating histone marks as negatives for the repressive histone marks and vice versa.)
2. Save about 0.5ug of chromatin for the **Total Input** sample. Store the reserved amount at -20ºC until the next day and then reverse the crosslinks in the input chromatin at the same time as the crosslinks in the ChIP samples are reversed.
3. Dilute chromatin 5-fold with ice-cold IP Dilution buffer (1 volume chromatin and 4 volumes IP Dilution buffer) containing protease inhibitors.
4. Add antibody specific to the histone mark of interest to capture the protein/chromatin complexes. ****Record amount of chromatin and antibody used and the catalogue number and lot number of antibodies for future reference!****

Antibody name	Antibody Provider	Antibody Cat#
H3K9Ac	Cell Signaling Technology<br>Millipore	9649s<br>07-352
H3K4me1	Diagenode	pAb-037-050
H3K4me3	Cell Signaling Technology	9751S
H3K9me3	Cell Signaling Technology<br>Diagenode	9754s<br>pAb-056-050
H3K36me3	Cell Signaling Technology<br>Diagenode	9763s<br>pAb-058-50
H3me3K27	Cell Signaling Technology	9733s

5. Incubate on a rotating platform at 4°C overnight.

Capture antibody/chromatin complex and reverse cross-links

1. Add 15 µl magnetic protein A/G beads to each ChIP sample (using from 1-5 ug chromatin/sample) and incubate on a rotating platform for 2 hrs at 4°C. We use an equal mixture of magnetic protein A beads from Cell Signaling Technology (#9006) and magnetic protein G beads from Diagenode (#AIP-102-015), but others work just as well. No carrier DNA should be added to beads.

****All the following steps are carried out at room temperature.****

1. Allow beads to settle for 1 min in a magnetic separation rack, then carefully remove the supernatant.
2. Wash magnetic beads two times with IP wash buffer 1 (RIPA buffer without protease inhibitors); take tubes out of magnetic rack and mix by pipetting. Efficient washing is critical to reduce background. Avoid cross contamination of samples and loss of magnetic beads.
3. Wash magnetic beads two times with IP wash buffer 2 (take tubes out of magnetic rack and mix by pipetting). Discard all wash solution after final wash.

4 Wash 1x with IP Wash buffer 3.

5. Elute antibody/chromatin complexes by adding 100 µl IP elution buffer. Shake on vortexer for 30 minutes, setting 4.
6. Allow beads to settle for 1 min in a magnetic separation rack. Carefully transfer supernatant containing antibody/chromatin complexes to a siliconized tube.
7. Add 12 µl of 5M NaCl (to give 0.6 M final concentration) to each tube. At this point, thaw the input sample from the previous day. Dilute 1 volume input sample with 4 volumes ChIP elution buffer (e.g. add 80 µl ChIP elution buffer to 20 µl input sample); add 12 µl of 5M NaCl per 100 µl elution buffer mix.
8. Incubate all samples in a 67°C water bath overnight to reverse formaldehyde cross-links.

DNA purification

1. Allow samples to cool down, add 1µl of RNaseA, incubate at 37°C for 20 min.
2. Purify DNA with a PCR clean up kit, one column per sample. Elute each sample with 30µl EB.
3. Assess ChIP enrichments by quantitative PCR (qPCR) before proceeding to preparation of Solexa libraries.

ChIP confirmation

Enrichment of histone marks in the ChIP samples are determined by quantitative real-time PCR (qPCR). The input sample is diluted with EB to give a final concentration of 2 ng/µl and serves as a

reference. Prepare a master reaction mix for each library with triplicate reactions per primer set. Add extra reagents for 10% of the total number of reagents to account for loss of volume. Add 14 µl of reaction mix to each PCR reaction well. Add 2 µl primer mix to each well.

Recipe for one reaction:

1µl	undiluted ChIP sample or diluted Input sample (2 ng/µl)
4.5 µl	Nuclease-free H20
7.5 µl	2X SYBR Green mix (containing polymerase)
2 µl	5 µM target primer mix (containing both Forward and Reverse primers)
15 µl	Total reaction volume

Amplify using the following PCR protocol:

3 min at 95ºC

40 cycles of 30 sec at 95ºC, then 30 sec at 60ºC

Include a 70-95°C melting curve at the end of the qPCR program, reading all points or every 0.2°C.

Analyze the qPCR results by first manually determining the cycle threshold for each reaction across the plate within the linear range of the amplification curve. Calculate the average cycle threshold for each triplicate reaction of each sample. The relative DNA amount is then calculated for any given primer set as 2 to the power of the cycle threshold (cT) difference between input chromatin and ChIP samples, where cT is the average value.

$$\text{Relative DNA amount} = 2\text{\^{}}(cT_{INPUT}\text{-}cT_{SAMPLE})$$

The enrichment is then calculated by comparing relative enrichment for the target and a negative control. This is accomplished by dividing the relative DNA amount of each sample for a target primer set by the corresponding value for a negative control primer set. The resulting quotient represents the fold enrichment.

Solutions for ChIP Assays

Protease inhibitor stock solutions (stored at -20ºC): Aprotinin (10 mg/ml), Leupeptin in water (10 mg/ml), 100 mM PMSF in isopropanol.
Note: Protease inhibitors and Igepal (NP-40) are added at time of use only. Do not add protease inhibitors to IP wash and elution buffers

Cell Lysis buffer
5 mM PIPES pH 8.0
85 mM KCl
Add Igepal (10 µl/ml), agitate at 37°C to dissolve, cool on ice
Add protease inhibitors [PMSF (10 µl/ml), aprotinin (1 µl/ml) and leupeptin (1 µl/ml)]

Nuclei Lysis buffer
50 mM Tris-Cl pH 8.1
10 mM EDTA
1% SDS
Add protease inhibitors [PMSF (10 µl/ml), aprotinin (1 µl/ml) and leupeptin (1 µl/ml)]

IP Dilution buffer (RIPA)
50 mM Tris pH7.4
150 mM NaCl
1 % (v/v) Igepal
0.25 % Deoxycholic acid
1 mM EDTA pH8
Add protease inhibitors [PMSF (10 µl/ml), aprotinin (1 µl/ml) and leupeptin (1 µl/ml)]

IP Wash buffer 1
50 mM Tris pH7.4
150 mM NaCl
1 % (v/v) Igepal
0.25 % Deoxycholic acid
1 mM EDTA pH8

IP Wash buffer 2
100 mM Tris-Cl pH 9.0
500 mM LiCl
1% Igepal
1% deoxycholic acid

IP Wash buffer 3
100 mM Tris-Cl pH 9.0
500 mM LiCl
1% Igepal
1% deoxycholic acid
150 mM NaCl

Elution buffer
50 mM NaHCO3
1% SDS

REMC Library Construction Protocol

The library protocol is based on the Illumina Sample Preparation Kit for Genomic DNA with some modifications. This protocol describes the preparation of libraries of ChIP DNA compatible with the Illumina sequencing platforms. Libraries are prepared from the ChIP sample as well as matching input DNA from the same cell type

Step 1: End-Repaire using "End-It DNA Repair Kit" from Epicentre (Cat# ER0720). This step ensures that all DNA fragments are converted to 5'-phosphorylated blunt-ended DNA.

A) The entire ChIP DNA volume is used. Combine and mix the following components in a siliconized eppendorf tube:
1-34 µl ChIP DNA or 200 ng input DNA
5 µl 10X End-Repair Buffer
5 µl 10 mM ATP
5 µl 2.5 M dNTP Mix
1 µl End-Repair Enzyme Mix
X ul sterile water to bring reaction volume to 50ul

B)Incubate at room temperature for 45 minutes and purify DNA using a PCR purification kit (such as QIAquick PCR purification kit). Elute in 34 µl EB.

Step2: Addition of 'A' base to 3' Ends

*Before starting, make up stocks of 1mM dATP using NEB 100mM dATP. (e.g. add 5ul of 100mM dATP to 495ul sterile RNase DNase free Gibco water; then make 25uL aliquots and freeze at -20C. You will want to freeze aliquots and defrost them only once).

A) Combine and mix the following components in PCR tubes:
34ul DNA from Step1
5ul Klenow buffer = NEB Buffer 2
10ul 1 mM dATP (you will have to make this up)
1ul Klenow fragment (3'→5' exo from NEB Cat# M0212s; 5,000 U/ml)
x ul sterile water to bring total reaction volume to 50 ul

B) Incubate for 30 minutes at 37°C (use PCR machine for incubation period).
C) Purify DNA using the **MinElute** PCR Purification Kit and protocol, and eluting in 11ul of EB.

Step 3: Adapter Ligation

*Dilute Illumina adapters 1:10 with water to adjust for the smaller quantity of DNA. Excess adapters can interfere with sequencing. Use diluted primers for one week and then discard.

PE Adapters

5' P-GATCGGAAGAGCGGTTCAGCAGGAATGCCGAG

5' ACACTCTTTCCCTACACGACGCTCTTCCGATCT

A) Combine and mix the following components in a siliconized eppendorf tube:

11ul DNA from Step2

15ul DNA ligase buffer

1ul Illumina Adapter oligo mix (1:10)

3 ul DNA ligase (Ligafast from Promega Cat# M8221)

total reaction volume = 30 ul

B) Incubate for 15 minutes at room temperature.

C) Purify DNA using a PCR purification kit (such as QIAquick PCR purification kit), elute in 19 µl EB buffer.

Step 4: Size Selection. Size selection of the sample ensures removal of unused adapters and selection of proper fragment size for amplification and sequencing. We use pre-cast agarose gels (Invitrogen E-Gel EX 2% agarose; Cat# G4020-02) to minimize risk of contamination.

A) Dilute 10 µl of 6x Cyan/Orange with 50 µl EB buffer to obtain 1x Cyan/Orange buffer dye. Add 1 µl of 1x Cyan/Orange buffer dye to eluted DNA from Step 3.
B) Load 20ul DNA with dye in each well (make sure to skip a well or two between each sample to avoid contamination of samples).
C) Load 20ul of 100bp ladder in the well marked "M."
D) Load 20uL of EB buffer in each of the empty wells.
E) Run the gel for 30 minutes.
F) Take a gel picture to visualize sample.
G) Size select libraries: using a clean razor blade, cut out 200-400bp bands and 400-600bp bands. (You may have to cut blindly due to low DNA concentrations).
H) Solubilize gels using QIAgen's gel extraction buffer at room temperature. Add 500uL QG buffer to each gel slice and shake on the vortexer for 30minutes.
I) Purify DNA using a QIAquick PCR Purification Kit. Elute in 25ul EB.

Step 5: Amplification of adapter-modified DNA fragments and gel purification.

Because we make libraries from both the small (200-400 bp) and the big (400-600 bp) size selected DNA fragments, we prepare 2 amplification reactions (and 2 libraries) per ChIP sample. We also prepare a 200-400 bp and a 400-600 bp size-selected input library. Therefore, if all 6 histone modifications are analyzed, there will be 14 amplification reactions and 14 libraries (12 ChIP libraries and 2 input libraries). Dilute Illumina paired-end primers (1.0 and 2.0) 1:4 using sterile water for use in the reactions. Paired end primers can be ordered via VMCS Oligo from companies such as Invitrogen. If possible, have primers go through HPLC check.

PE PCR Primer 1.0

5' AATGATACGGCGACCACCGAGATCTACACTCTTTCCCTACACGACGCTCTTCCGATCT

PE PCR Primer 2.0

5' CAAGCAGAAGACGGCATACGAGATCGGTCTCGGCATTCCTGCTGAACCGCTCTTCCGATCT

A) Combine and mix the following components in PCR tubes:

23ul DNA from Step 4

25ul Phusion DNA polymerase

1ul PCR primer 1.0 (diluted 1:4)

1ul PCR primer 2.0 (diluted 1:4)

50ul Total reaction volume

B) Amplify using the following PCR protocol:

- 30 seconds at 98C
- [10 seconds at 98C, 30 seconds at 65C, 30 seconds at 72C] for 15 cycles
- 5 minutes at 72C
- Hold at 4C

Start with 15 cycles of amplification; 5 additional cycles can be added if libraries are not quantifiable in step 6. However, over-amplification should be avoided to reduce the risk of PCR artifacts.

Step 6: Library Purification

Use Agencourt Ampure, Beckman Coulter (Cat# A29152) to purify library samples.

A) Mix Ampure beads thoroughly before addition.

B) Add 90ul of Ampure beads to each 50ul DNA sample from Step 5. Pipette several times to ensure proper mixing.

C) Use magnetic stands to separate bead-DNA complexes and discard supernatant. (It may take several minutes for all the beads to separate from the supernatant).

D) Wash bead-DNA complexes using 70% ethanol; when washing, be sure not to disturb the beads. Leave the tube in the magnetic stand and add 200ul of 70% ethanol. After 30 seconds, discard 70% ethanol. Repeat for a total of two washes.
E) Allow beads to dry. This may take anywhere from 10-20 minutes.
F) Add 30ul of EB buffer and shake on the vortexer for 30 minutes.
G) Place tubes back in magnetic stands to collect DNA; transfer liquid to siliconized Eppendorf tubes. Store libraries at -20ºC

Step 7: Library Quantification and Confirmation

To verify that a ChIP library has maintained a specific enrichment of target sites, perform qPCR on the ChIP-seq library using both positive targets and negative control primer pairs. The input library serves as a control to normalize the qPCR data to determine the relative enrichment of a given target.

Real-time quantitative PCR (qPCR)

Analyze the ChIP-seq sample as well as the appropriately sized input library for reference. Prepare a master reaction mix for each library with triplicate reactions per primer set. Add extra reagents for 10% of the total number of reagents to account for loss of volume. Add 15 µl of reaction mix to each PCR reaction well. Add 2 µl of primer mix to each well.

2µl	2 ng library
3.5 µl	Nuclease-free H20
7.5 µl	2X SYBR Green mix (containing polymerase)
2 µl	5 µM target primer mix
15 µl	Total reaction volume

Amplify using the following PCR protocol:

3 min at 95ºC

40 cycles of 30 sec at 95ºC then 30 sec at 60ºC

Include a 70-95°C melting curve at the end of the qPCR program, reading all points or every 0.2°C.

Analyze library enrichments by qPCR as described above. Do not proceed with sequencing unless the positive targets are at least 20 fold enriched.
