# Supplementary figures and images for: Characterization of the Contradictory Chromatin Signatures at the 3′ Exons of Zinc Finger Genes
Source: PLoS One. 2011 Feb 15;6(2):e17121. doi: 10.1371/journal.pone.0017121 (PMC3039671; doi:10.1371/journal.pone.0017121)

A

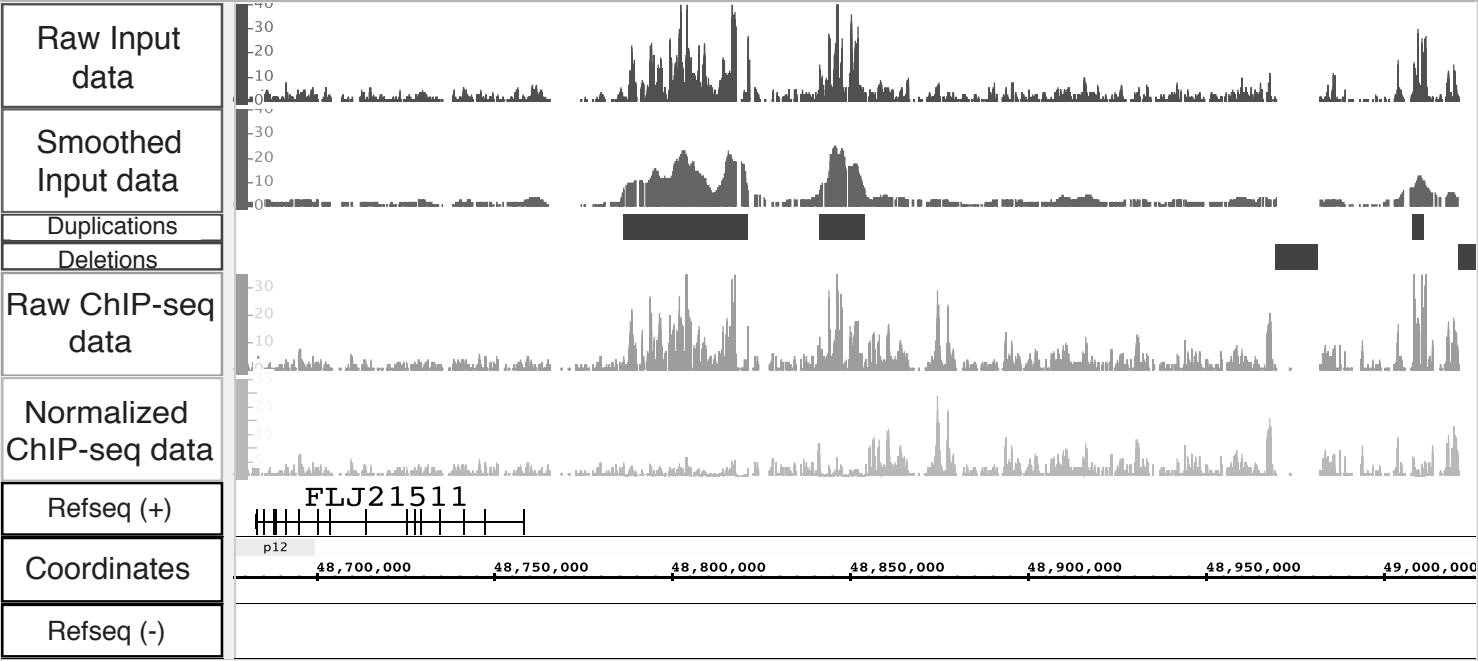

B

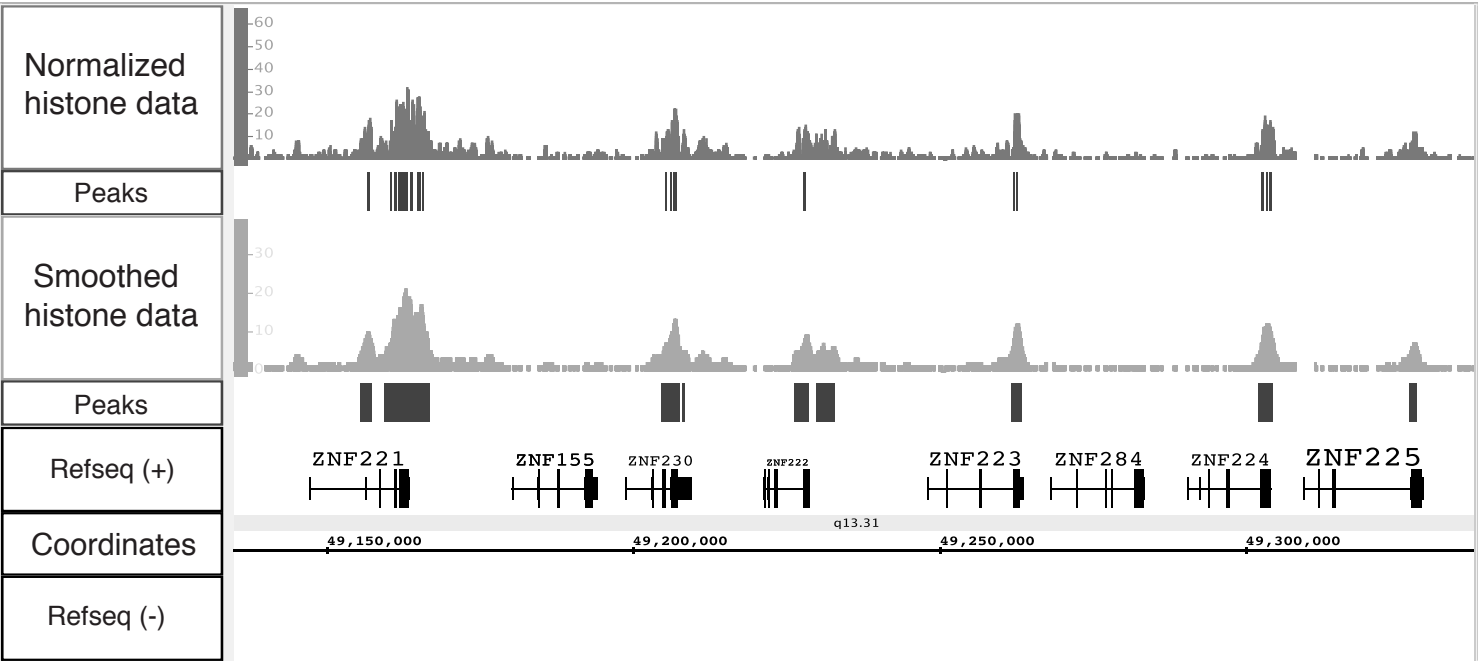

C

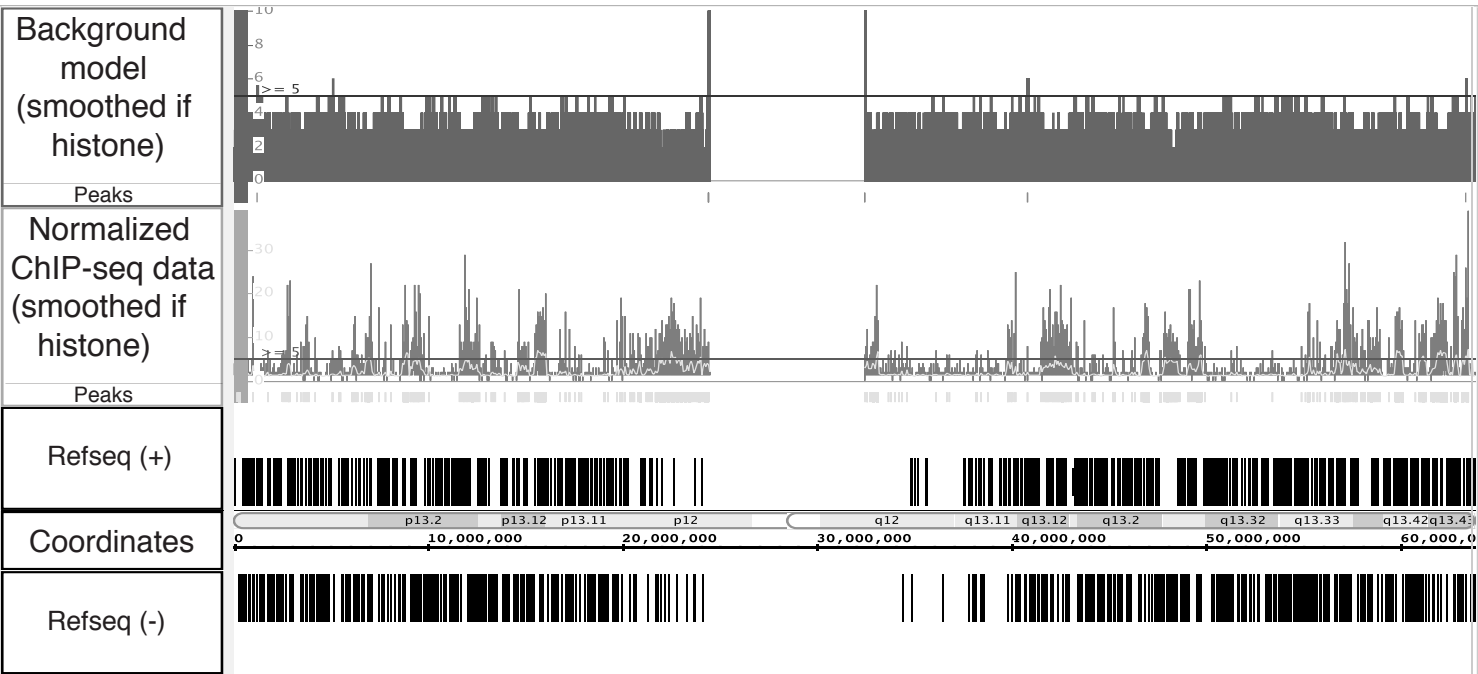

Supplement: Figure S1 — Major steps implemented in Sole-searchv2. The original Sole-search program has been modified to improve the ability to determine statistically and biologically significant peaks in both transcription factor and histone modification datasets. (A) Input data is smoothed and duplication and deletion events are determined based on fold coverage of these regions compared to average coverage. Next, specific regions of the genome that have a higher sequence coverage than expected by chance, due to experimental method, are determined using a t-statistic. Raw ChIP-seq data is normalized based on duplication event copy number and enrichment of input reads, so that the data to be analyzed will reflect a single copy genome without sequencing bias (see the track corresponding to “Normalized ChIP-seq data”). (B) The second, optional step smoothes data that spreads over large regions (e.g. data from histone modification ChIP-seq experiments), using a sliding average, so that non-uniform “mountain range” peaks are more easily detectable as broad regions. Previously, these regions would be identified as having many, distinct peaks. Also certain smaller peaks would fail to be detected. This smoothing step allows detection of both broad regions and smaller. (C) The third major step determines a statistically significant peak height cutoff. Tags are randomly sampled from the input dataset to create bins. Tags are then shuffled within the bins. Height cutoff is determined based on a user-defined FDR. The cutoff value increases until the number of peaks found within the randomly generated background is sufficiently low, compared to the number of peaks found in the ChIP-seq dataset at the same height. (PDF) [file pone.0017121.s002.pdf]

Figure S2

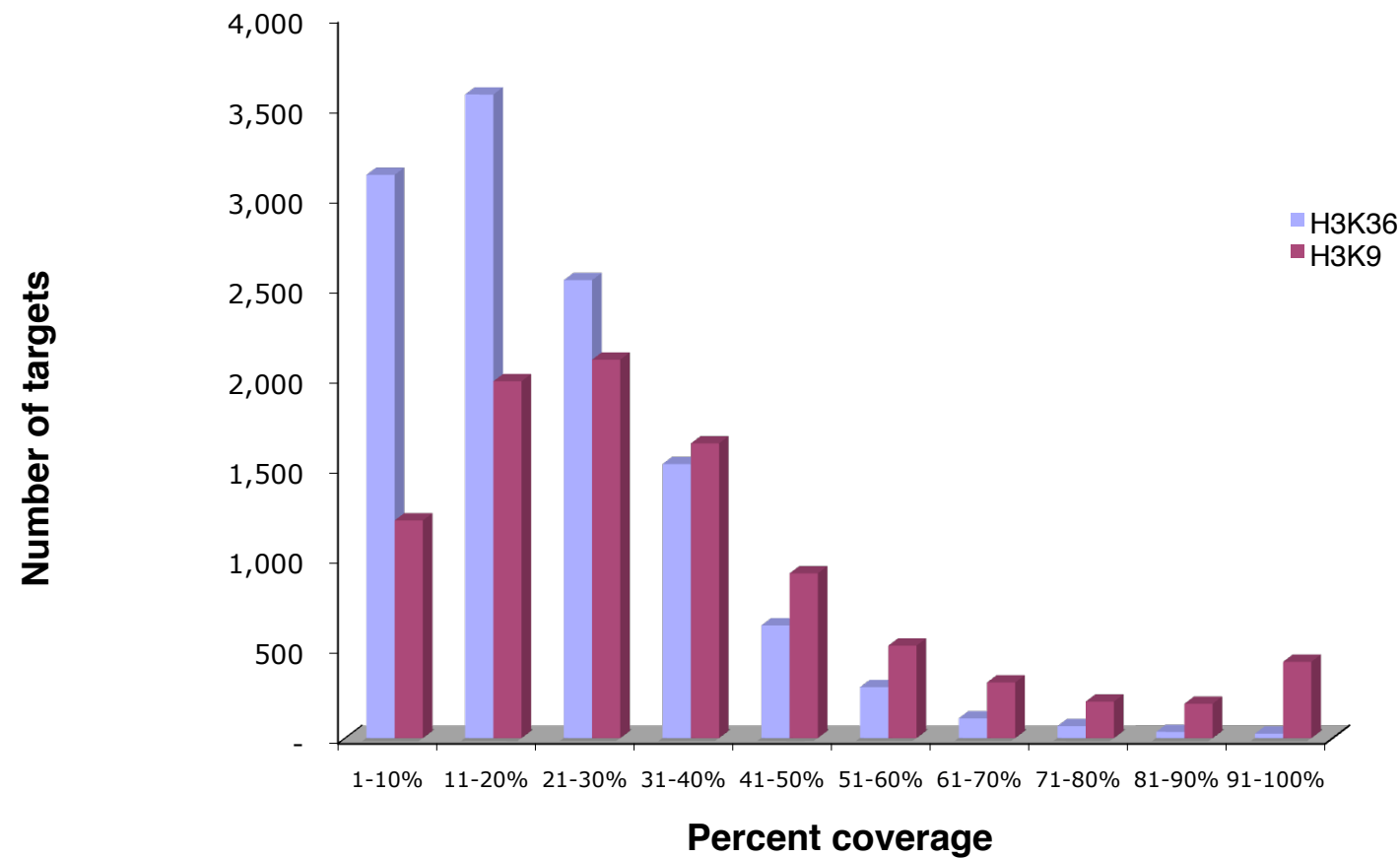

Supplement: Figure S2 — 20744 H3K9me3 peaks and 20744 H3K36me3 peaks were searched for tandem repeats using the program XSTREAM ( http://www.ncbi.nlm.nih.gov/pmc/articles/PMC2233649/ ), identifying sequences that are 50bp or larger, repeated at least 5 times in the human genome, with at least 60% conservation between repeat elements. The percent of each peak that was a repetitive element was then calculated. A greater number of the H3K9me3 peaks had high percentages of repetitive regions. For example, there are ∼17 times more H3K9me3 peaks that consist of 91-100% repetitive elements. (PDF) [file pone.0017121.s003.pdf]
