# Supplementary material for: Characterization of the Contradictory Chromatin Signatures at the 3′ Exons of Zinc Finger Genes
Source: PLoS One. 2011 Feb 15;6(2):e17121. doi: 10.1371/journal.pone.0017121 (PMC3039671; doi:10.1371/journal.pone.0017121)
Supplement: Table S1 — Summary table of ChIP-seq data sets. (PDF) [file pone.0017121.s004.pdf]

Table S1: Summary table of ChIP-seq data sets

Cell type	ChIP	Replicate	Library	Unique reads	genome	Project
H1hES	H3K9me3	cd1	PF004/HS0998	8,794,834	hg18	REMC
	H3K36me3	cd1	PF006/HS1032	12,726,604	hg18	REMC
	H3K4me3	cd1	HS0996 (PF002)	3,854,913	hg18	REMC
Ntera2	H3K9me3	Replicate 1	UCD319	58219858	hg19	ENCODE
	H3K9me3	Replicate2	UCD389	36261556	hg19	ENCODE
	H3K36me3		UCD320	38312545	hg19	ENCODE
K562	H3K9me3		UCD393	18,842,354	hg19	ENCODE
	H3K36me3		UCD394	18,935,118	hg19	ENCODE
U2OS	H3K9me3		UCD339	15,596,716	hg19	ENCODE
	H3K36me3		UCD401		hg19	ENCODE
TC010 PBMC	H3K9me3		HS2622	19,828,553	hg18	REMC
	H3K36me3		HS2624	23,776,023	hg18	REMC
