# Supplementary material for: Characterization of the Contradictory Chromatin Signatures at the 3′ Exons of Zinc Finger Genes
Source: PLoS One. 2011 Feb 15;6(2):e17121. doi: 10.1371/journal.pone.0017121 (PMC3039671; doi:10.1371/journal.pone.0017121)
Supplement: Table S2 — List of primers used in this study. (PDF) [file pone.0017121.s005.pdf]

Blahnik_Table S2

Gene	Genome	Chromosomal coordinates
ZNF440 promoter	hg17	chr19:11785623-11786200
ZNF554 promoter	hg17	chr19:2770497-2771257
ZNF440 3' end	hg17	chr19:11804214-11804933
ZNF554 3' end	hg17	chr19:2784992-2785996
ZNF7 3' end (AF027146)	hg17	chr8:146037574-146038214
ZFP1 3' end (BC033774)	hg17	chr16:73760379-73761021
ZFP3 3' end (AK125731)	hg17	chr17:4935767-4936411
ZNF473 3' end (AL080143)	hg17	chr19:55240500-55241079
ZNF79 3' end (X65232)	hg17	chr9:127286029-127286631
ZNF33' end (AF027139)	hg17	chr7:99313806-99314437
ZNF555 3' end endogenous	hg19	chr19:2853275-2853474
ZNF556 3' end endogenous	hg19	chr19:2877750-2877960
ZNF77 3' end endogenous	hg19	chr19:2933551-2933800
ZNF333 3' end endogenous	hg19	chr19:14829471-14829751
ZNF426 3' end endogenous	hg19	chr19:9639938-9640235
GAPDH endogenous	hg19	chr12:6645894-6646027
GMNN 5' end endogenous	hg19	chr6:24774769-24774958
ZNF555 3' end episome	hg19	chr19:2852308-2853885
ZNF556 3' end episome	hg19	chr19:2877226-2878368
ZNF77 3' end episome	hg19	chr19:2933208-2934672
GMNN episome	hg19	chr6:24774850-24775349
episome vector forward	pFC62-F	GGCAACAGACAGGTCTGACA
episome vector reverse	pFC62-R	AAACATCCCCGGGTACTGG
Hygromycin (episome) forward	hyg 3'-F	GAGTGCTGGGGCGTCGGTTTCC
Hygromycin (episome) reverse	hyg 3'-R	CCGCATTGGTCTTGACCAAC
ZNF799 3' end	hg17	chr19:12363548-12363658
ZNF420 3' end	hg18	chr19:42310770-42311115
ZNF682 3' end	hg18	chr19:19978299-19978513
ZNF556 3' end	hg19	chr19:2877226-2878368
GMNN 5' end	hg19	chr6:24774769-24774958
